# Engineering and Application of a Thermostable MHETase for PET Depolymerization

**DOI:** 10.1021/acssuschemeng.6c01404

**Published:** 2026-06-08

**Authors:** Natasha P. Murphy, Japheth E. Gado, Tabea Neumann, Pablo Perez-Garcia, Evan Komp, Luisana Avilan, Irimpan I. Mathews, Elizabeth L. Bell, Brenna Norton-Baker, Matilda Clark, Rebecca R. Garcia, Hannah M. Alt, Ritimukta Sarangi, Andrew R. Pickford, Wolfgang R. Streit, John E. McGeehan, Nicholas P. Gauthier, Gregg T. Beckham

**Affiliations:** † Renewable Resources and Enabling Sciences Center, National Laboratory of the Rockies, Golden, Colorado 80401, United States; ‡ BOTTLE Consortium, Golden, Colorado 80401, United States; § Agile BioFoundry, Emeryville, California 94608, United States; ∥ Department of Microbiology and Biotechnology, University of Hamburg, Hamburg 22609, Germany; ⊥ Centre for Enzyme Innovation, School of the Environment and Life Sciences, 6697 University of Portsmouth, Portsmouth PO1 2DY, U.K.; # SLAC National Accelerator Laboratory, Stanford Synchrotron Radiation Lightsource, Menlo Park, California 94025, United States; ∇ Department of Systems Biology, 1811 Harvard Medical School, Boston, Massachusetts 02115, United States; ○ Department of Data Sciences, Dana-Farber Cancer Institute, Boston, Massachusetts 02115, United States

**Keywords:** Enzymes, High-throughput assay, Interfacial biocatalysis, Polymers, Protein engineering

## Abstract

Enzymatic hydrolysis of poly­(ethylene terephthalate) (PET) releases mono­(2-hydroxyethyl) terephthalate (MHET) as a major product, the accumulation of which can prolong reactor residence times and complicate downstream monomer separations. The use of a MHETase enzyme can enable MHET hydrolysis to the monomers, terephthalic acid and ethylene glycol, but industrial PETases typically operate at thermophilic temperatures and the well-known MHETase from *Ideonella sakaiensis* is a mesophilic enzyme, thus warranting the development of thermophilic MHETases. Here, we characterize thermostable MHET-active enzymes from a natural diversity screen by applying a hidden Markov model based on the previously reported, archaeal ferulic acid esterase, PET46. We identified enzymes with higher thermostability than PET46 and quantified their MHETase activity in reactions at 70 °C. The crystal structure of MHT077, the homologue with the highest MHETase activity and an apparent melting temperature (*T*_m,app_) of 94.6 °C, informed site saturation mutagenesis in the active site and lid-domain interface. MHT077 exhibited a ∼100-fold slower unfolding rate at 65 °C than PET46, indicating substantially greater kinetic stability. In parallel, we applied evolution-informed design, a probabilistic model that leverages coevolutionary patterns in large multiple sequence alignments, to improve the activity and thermostability of five ferulic acid esterases. One design, EV-MHT043–5 was identified with a comparable thermostability (*T*_m,app_ = 96.1 °C) and a 3-fold improvement in its MHETase activity relative to the wildtype enzyme, MHT043. Combination variants of beneficial mutations were screened and afforded a variant, MHT077^LFK^, which reduced MHET accumulation in bioreactor experiments with postconsumer PET waste. Overall, this study expands the known MHET-hydrolyzing protein scaffolds available for enzymatic PET recycling.

## Graphical abstract

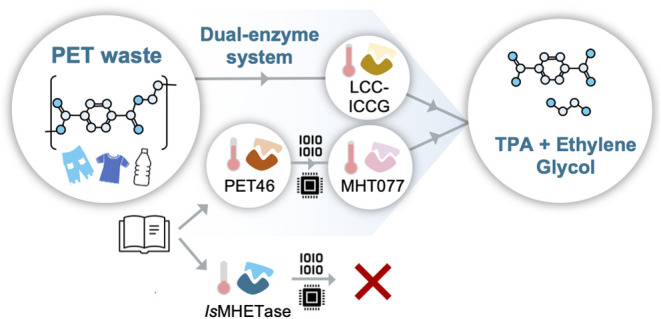

## Introduction

Hydrolase enzymes are promising biocatalysts for chemical recycling of poly­(ethylene terephthalate) (PET), the most ubiquitous synthetic polyester.^1–4^ A two-enzyme system, PETase and MHETase, was previously reported in *Ideonella sakaiensis* to cleave the ester bonds of PET and the intermediates, bis­(2-hydroxyethyl) terephthalate (BHET) and mono­(2-hydroxyethyl) terephthalate (MHET), to the monomers ethylene glycol and terephthalic acid (TPA).^5,6^ In the last several years, PET hydrolases that are thermostable and efficiently degrade PET have been the subject of intense engineering and landscape-profiling efforts.^7–12^ Thermostable PET hydrolases, either those derived from thermophilic microorganisms or engineered for thermostability, efficiently function at temperatures significantly higher (50–70 °C) than *Is*PETase (∼30 °C) and typically exhibit apparent melting temperatures (*T*_m,app_) above 80 °C.^11,13–18^ As a significant portion of products released by PET hydrolase activity are oligomeric intermediates,^19^ MHET accumulates during reactor-scale enzymatic PET degradation,^20,21^ leading to reduced TPA yields and extended residence times. Stable and durable MHETases with high thermostabilities could therefore be beneficial for industrial applications to synergize with PETase-driven industrial processes in two-enzyme systems.^22–24^

Candidate enzymes for MHET hydrolysis have to date been sourced from a variety of enzymes classes that catalyze ester hydrolysis including tannases,^22^ carboxylesterases,^25^ and ferulic acid esterases (FAEs).^8^ The best-characterized example with annotated MHET hydrolase function is *Is*MHETase, which belongs to the tannase family (InterPro ID: IPR011118), has a *T*_m,app_ of ∼51 °C, and optimal activity between 30 and 45 °C.^22,26^ Engineered variants of *Is*MHETase and its homologues,^27,28^ show potential for use in two-enzyme systems that operate above 60 °C.^22,24,26,29–31^ Several thermostable carboxylesterases with hydrolase activity against MHET, BHET, and PET oligomers, have also been discovered and engineered for dual-enzyme systems.^32–36^ Computational methods that incorporate evolution-informed design have also aided the development of thermostable enzymes by applying evolutionary models that account for residue conservation and pairwise interactions across diverse sets of homologous sequences.^37–39^ Concurrently, genomic mining of extremophiles has yielded thermostable biocatalysts capable of soluble ester hydrolysis.^8,25,40,41^ Thermostable FAEs such as PET46, derived from a deep-sea archaeal strain, *Candidatus* Bathyarchaeota, can hydrolyze both BHET and MHET.^8,42^ While enzymes such as PET46 with a narrow active site covered by a lid are likely structurally limited in their ability to accommodate longer-chain PET substrates and therefore display sparing PET hydrolysis activity,^8,43^ they represent evolvable starting scaffolds for MHET hydrolysis. Compared to the large ∼240-amino acid lid domain of *Is*MHETase that is essential for catalysis,^22^ PET46 has a significantly smaller and structurally compact, 40-amino acid lid domain.^8^

In this work, we hypothesized that an esterase active site covered with a considerably smaller lid domain than that of *Is*MHETase would be a promising feature for engineering thermophilic MHET degradation. As PET46 exhibits a high thermostability (*T*_m,app_ *=* 88 °C), we considered it an excellent starting point to expand the diversity of MHET hydrolase enzymes that could operate at elevated temperatures. A natural diversity screen using a hidden Markov model based on PET46 identified six enzymes with higher MHET hydrolase activity than both *Is*MHETase and PET46 at 70 °C. The crystal structure of the most active homologue, MHT077, a thermostable FAE (*T*_m,app_ = 94.6 °C), was used to inform site-saturation mutagenesis of residues in the lid domain and active-site interface. A resulting variant, W148F, showed a 3.7-fold increase in MHET conversion at 70 °C and was subsequently combined into double and triple mutants for further enhancements. In parallel, using evolution-informed design, we identified variants with improvements in durability and activity. Overall, this study provides insights that can be used to guide future protein engineering efforts to enhance the efficiency of thermostable enzymes with MHET hydrolase activity.

## Results and Discussion

### Hidden-Markov Model Sequence Search to Identify Putative Thermophilic MHETases

To expand the known diversity of FAEs and identify potentially thermostable MHETase scaffolds with small, lid-domain covered active sites, we initially used a hidden Markov model approach, based on the previously reported archaeal PETase, PET46.^8^ The MGnify and NCBI nr databases were downloaded on May 23, 2023,^44,45^ to a local cluster, and a phmmer search against these databases was conducted using PET46 as the query sequence and a bitscore threshold of 100 using the hmmer software (v3.1b2).^46^ We chose phmmer for this analysis instead of hmmsearch, commonly used in similar studies,^43,47^ because the low homology and poor alignment of PET46 with other known MHETases made constructing a reliable profile HMM with hmmbuild unfeasible. The phmmer search identified 5,067 sequences, most with low bitscore (median 113.8, max of 580.6). *T*_m_ values were predicted using thermal stability predictor (ThermoPalm^12^), trained with a ridge regression model on mean-pooled ProtT5-XL-U50 embeddings and melting temperature data from the Meltome Atlas.^48,49^ Optimal pH (pH_opt_) was predicted using EpHod.^50^ Sequences were percentile-ranked based on their phmmer alignment scores, predicted thermostability, and predicted pH_opt_ values, from lowest or least acidic (0) to highest or most acidic (100), to prioritize variants with potentially enhanced activity and thermostability under acidic conditions. The phmmer alignment score was used as a proxy for predicted MHETase activity, based on the assumption that higher homology to PET46 corresponds to a greater likelihood of MHETase activity.

To select promising sequences, we identified those with phmmer, *T*_m_, or acidic pH_opt_ scores in the top 2% (69 sequences) as well as those with all three scores in the top 20% (87 sequences), yielding 147 sequences in total. Sequences were selected for acidic pH_opt_ scores, as shifting the pH optimum of MHETases closer to neutral (i.e., toward pH 6–7) may improve compatibility with industrial-scale process conditions for the application of a coupled PETase-MHETase system (PET46 was predicted to have a pH_opt_ of 8.14 using the EpHod model). All sequences were clustered at 95% identity using mmseqs2, forming 101 clusters. From each cluster, the sequence with the highest percentile score in any of the three metrics were selected, resulting in 94 candidates. We manually inspected the selected sequences and excluded spuriously long sequences with additional flanking domains and sequences poorly aligned to the PET46 catalytic domain. The resulting 94 candidates are shown in addition to 41 MHETase sequences mined from the literature (Figure 1A, gray and black dots, Figure S1).

**Figure 1:**
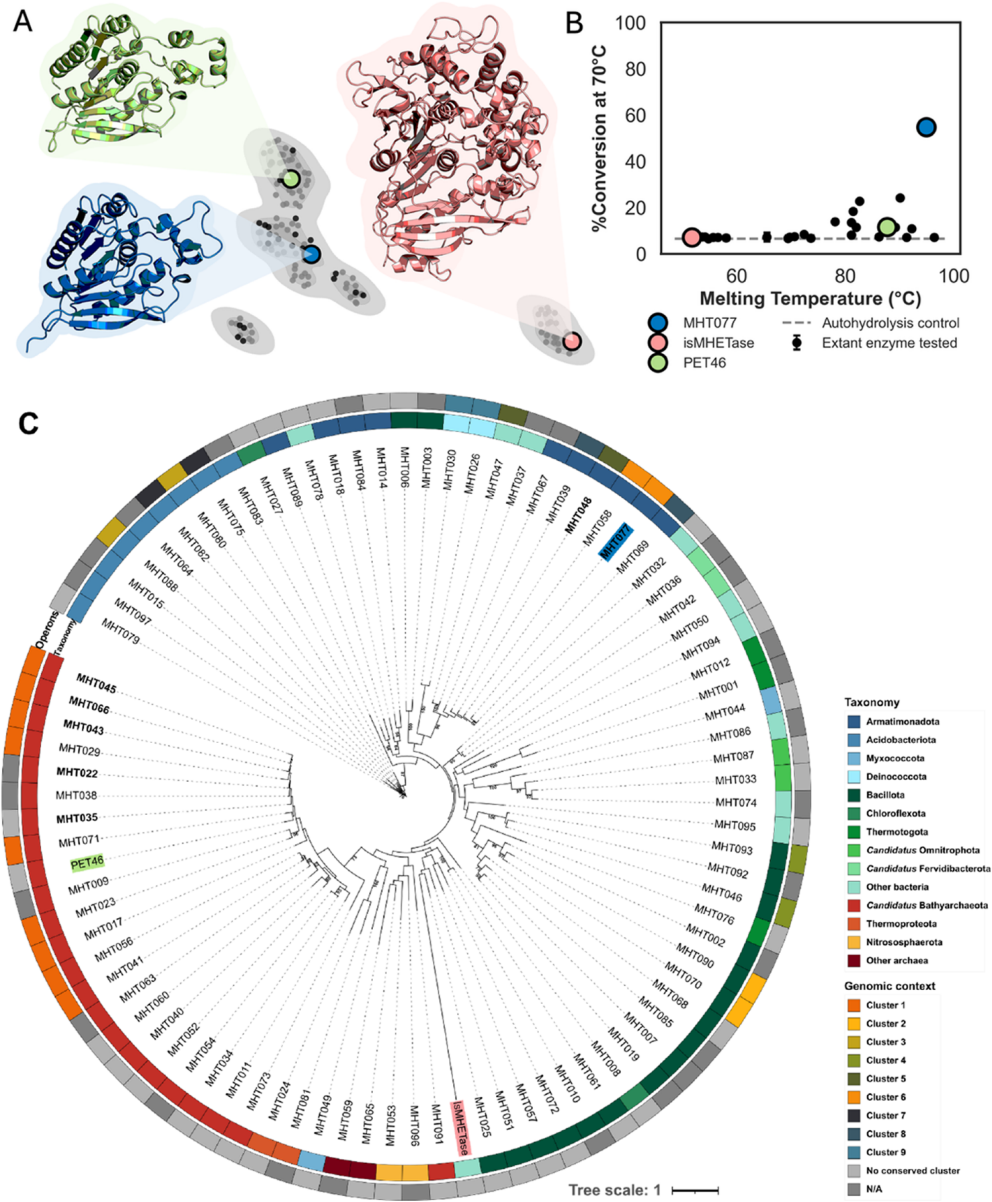
Putative MHETase enzymes explored in this study. (A) Structural diversity of 94 putative MHETases tested in the context of 41 previously studied MHETases. Three enzymes are highlighted–*Is*MHETase (pink), PET46 (green), and MHT077 (blue), the best-performing wild type enzyme identified in this study. Structure space was determined by UMAP^56^ (min distance 0.8, N. neighbors 20) of pairwise negative log local distance difference test (LDDT) values. LDDT was computed using the Foldmason alignment^57^ (10 refinement iterations) of AlphaFold3 (AF3)^58^ predicted structures, with LDDT for equivalent residue α carbons at Å-cutoffs 0.5, 1.0, 2.0, and 4.0 (max distance 15 Å) averaged over all alignment positions. Positions where one but not both proteins had matches in the alignment were counted as 0.0 and double gaps ignored. Candidates in (B) are shown in black while others are gray.(B) Experimentally measured MHETase activity shown as percent conversion at 70 °C and thermal stability of select enzymes including the same three highlighted in panel (A), with MHT077 having the highest MHET conversion and apparent melting temperature. The dashed line indicates the autohydrolysis conversion of the MHET substrate control to TPA in 100 mM phosphate pH 8. (C) Maximum likelihood phylogenetic tree of PET46, *Is*MHETase, and 87 MHT sequences. Active sequences in this study are highlighted in bold; *Is*MHETase, PET46, and MHT077 are displayed in the same colors as in panels (A, B). Sequences were aligned using Clustal Omega.^59^ Phylogenetic analysis was performed with RAxML-NG,^60^ employing the LG substitution matrix, 8 discrete γ categories, and 10 randomized parsimony starting trees. Node support was assessed using 200 bootstrap replicates; only values ≥70% are shown. Taxonomy was assigned based on the best hit from a DIAMOND v.2.0.15 ^61^ BLASTp search against the NCBI nr database. More information on clustering according to genetic context is provided in Figure S3. Tree visualization was done in iTOL v6.^62^

The set of 94 sequences were expressed in *E. coli*, purified, and screened for both thermostability and MHETase activity using high-throughput, robot-assisted protocols^51^ (Figure 1B). All purified enzymes were normalized in concentration to 0.1 mg/mL prior to screening (2 mM MHET in 0.1 M NaPi pH 8, 1 h, 70 °C). All enzymes were preincubated at 70 °C for 2 h, prior to a 1 h reaction. MHETase activity was determined as a percent MHET conversion, the percentage of initial MHET hydrolyzed to TPA, In quantified by ultrahigh performance liquid chromatography (UHPLC). The 29 sequences with appreciable MHETase activity at 70 °C in the screen, in addition to PET46 and *Is*MHETase, are displayed in Figure 1B as black dots. We observed three distinct structural nodes, and found MHETases active at 70 °C in two nodes, with sequence identities between active enzymes as low as 21%. Six enzymes were identified with a higher MHETase activity (calculated as % MHET conversion) than PET46 at 70 °C (Figure 1B). At 70 °C, after 1 h of incubation, MHT077 displayed a 54.7 ± 1.5% conversion compared to 10.1 ± 0.4% by PET46. All enzymes were also assayed for their activity at 50 and 60 °C (Figure S2).

The most active homologue identified, MHT077, is a 252-amino acid protein with a dienelactone hydrolase superfamily domain and 37% sequence identity to the FAE PET46 (RLI42440.1). To better understand the diversity of all sequences, we analyzed their taxonomic origin, revealing a broad distribution across archaeal and bacterial lineages. Among 94 candidates, we identified 29 archaeal sequences, predominantly from *Candidatus* Bathyarchaeota, including PET46 and active homologues MHT022, MHT035, MHT043, MHT045, and MHT066. The most active bacterial homologues, MHT048 and MHT077, originate from the Armatimonadota phylum (Figure 1C), a sparsely characterized group with some members previously isolated from hot springs and other (hyper)­thermophilic environments,^52,53^ and with genomic features supporting conserved polysaccharide metabolism.^54^

To investigate the genomic context of these MHT genes, we retrieved the original contigs and found up to nine distinct genetic neighborhood clusters. In archaeal cluster 1 (10 contigs), MHT candidates were consistently located downstream of an MBL-fold metallo-hydrolase, known for their substrate promiscuity,^55^ with adjacent genes frequently related to nutrient uptake or cell wall synthesis (Figure S3). MHET-active Armatimonadota homologues MHT048 and MHT077 were assigned to clusters 5 and 6, respectively, with cluster 5 featuring genes involved in nucleic acid and toxin-antitoxin regulation, and cluster 6 enriched in functions related to secondary metabolism, aromatic compound degradation, and detoxification (Figure S3).

### Structural Analyses of Active MHET Hydrolases

The three-dimensional structure of MHT077 was determined by X-ray diffraction at 1.15 Å resolution (PDB: 9PIC, Figure 2A,B and Table S1) and revealed a conserved protein fold comprising a canonical eight-strand α/β- fold architecture, with a lid domain of 40 amino acids (N144–K178) that confines the catalytic residues, S117, H229, and D198. The structures of the other active homologues were predicted using AF3.^58^ All active homologues share a highly conserved eight-strand α/β-fold architecture, common to PET46 (Figure 2A,B), in particular around the active site, where the relative positions of the active site triads are almost identical (Figure 2C). Similarly, the narrow active site cavity is retained across the homologues (Figures 2A,B and S4), although there are some differences in the distribution of electrostatic potential on surfaces distal from the active site (Figure S4). Structural comparisons revealed small differences in the lid domain region among the homologues (Figure S5). Relative to PET46 (PDB: 8B4U),^8^ MHT035, MHT043, MHT045, and MHT066 retained the predicted secondary structure elements with three short α-helices and two antiparallel β-strands. In contrast, MHT014, MHT048, and MHT077 display increasing divergence from the PET46 fold, exhibiting a loss of secondary structure. Comparison of the X-ray structures of PET46 and MHT077 reveals higher B-factors for lid domain residues in MHT077 and may indicate higher mobility of this region compared to the enzyme core (Figure S6).

**Figure 2:**
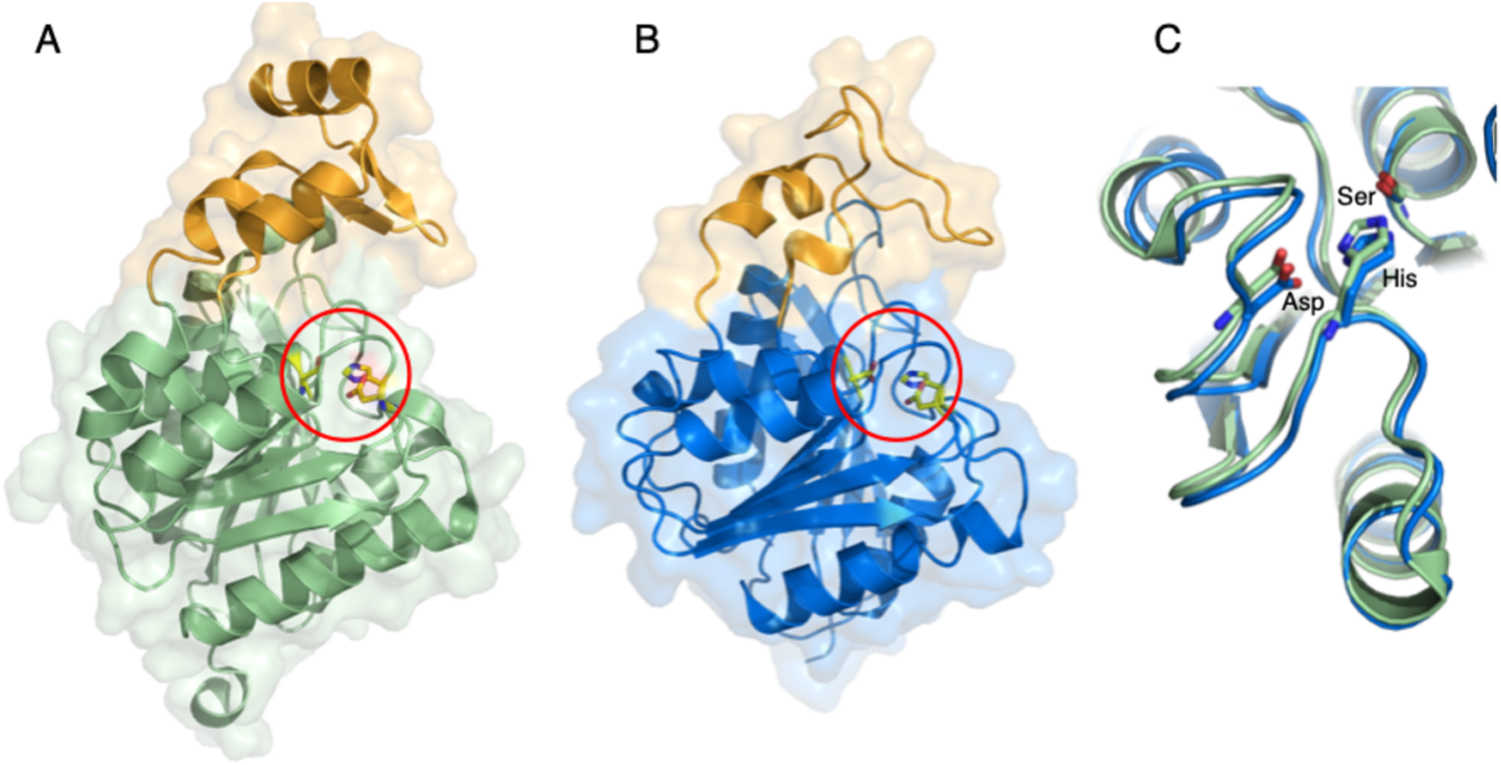
Structural comparison of PET46 and MHT077. Cartoon and transparent surface representations of (A) PET46 (PDB: 8B4U)^8^ and (B) MHT077 (PDB: 9PIC) reveal minor rearrangements in the core domain, with the active sites highlighted by a red circle. As shown in orange in both structures, a comparative loss of strongly predicted secondary elements is observed in the MHT077 lid, whereas the PET46 lid has three α-helices and a pair of antiparallel β-strands. (C) The surrounding topology of the active site of the two enzymes is highly conserved, resulting in almost identical arrangements of the active site triads between PET46 (Ser115, His238, and Asp206) and MHT077 (Ser117, His229, and Asp198), with catalytic residues represented as sticks.

The thermal durability of MHT077 was characterized using multiple scan rate differential scanning calorimetry (MSR-DSC) and compared to PET46, where the unfolding of both was established to be an irreversible process from the native to denatured state (Figures S7–S8 and Tables S2–S3). The greater susceptibility to denaturation of PET46 compared to MHT077 was evident in its lower apparent melting temperatures (Table 1), which lie approximately at the maximum heat capacity (*C*_p_) value of each thermal profile (Figure S7). The rates of unfolding over time of PET46 and MHT077 were calculated at 65 °C (Table 1), the operational temperature of benchmark thermostable PETase, LCC^ICCG^.^14,63^ A ∼100-fold slower rate of unfolding of MHT077 relative to PET46 confirmed its higher kinetic stability. The pH-activity profiles were also characterized by MHET hydrolysis assays across single integer intervals of pH ranging across 2 to 9. Both PET46 and MHT077 exhibited the highest activity at pH 8, with a complete loss of activity at pH 2–3 (Figure S9). The experimentally measured optimal pH values were therefore consistent with those predicted by EpHod for both PET46 (pH 8.16) and MHT077 (pH 7.85).

**Table 1:** Calculated Rates of Unfolding of PET46 and MHT077 at 65 °C^a^,^b^,^c^,^d^,^e^ ^64^

Enzyme	*E*_a_ (kJ/mol)^e^	T* (°C)^e^	*T*_m,app_ (°C)^e^	*k* at 65 °C (s^–1^) (×10^–8^)^e^
PET46	428 ± 1.0	95.2 ± 0.1	78.8–85.4	373 ± 12
MHT077	526 ± 1.5	99.3 ± 0.1	85.7–91.7	3.3 ± 0.2

a Energetic analyses of MSR-DSC thermograms applied an irreversible, native-to-denatured kinetic model using CalFitter v2.0.^64^

b The activation energy for unfolding, where errors indicate the parameter confidences calculated by CalFitter v2.0.

c The temperature at which the kinetic rate constant for the native-to-denatured transition (*k*) is 1 s^–1^.

d The range of apparent *T*_m_ values observed across the scan rate window of 0.2–3.2 °C/min, respectively.

e Calculated from *E*_a_ and T* using the Arrhenius equation (Experimental section).

### Structure-Informed Site-Saturation Engineering of MHT077

Given that MHT077 is an improved MHETase scaffold, engineering efforts were next directed toward both the active-site and lid-domain regions of the enzyme. Attempts made to cocrystallize MHT077 with MHET or substrate analogues (MHET-amide and BHET) were unsuccessful. Following the observation that ethylene glycol appeared to be occupying the active site pocket and binding through Glu47, we generated an E47A variant that retained activity and crystallized it (PDB: 9PIR), but we were unable to form a ligand-bound complex (Figure S10). Using the crystal structure of MHT077, MHET docking was performed with AF3 ^58^ and NeuralPLexer,^65^ but neither yielded convincing substrate geometries relative to the active site triad.^22^ Manual docking was performed using ligand-bound structures of FAE (7Z2U)^66^ and cinnamoyl esterase (3S2Z)^67^ as templates for MHET orientation relative to their highly homologous active site triads (Figure 3A). Further docking focusing on the position of the aromatic ring of the bound ligands (ferulate and caffeic acid) in these homologues suggests that residue W148 sterically impedes a productive orientation of MHET (Figure S11). While this residue may reorientate through MHET induced-fit binding, it presented an obvious target for mutagenesis. Thus, residue W148, was targeted and a crystal structure was determined for one these variants, MHT077^W148R^ (PDB: 9PID). Structural comparisons show no significant structural differences from the parent enzyme MHT077, with an RMSD of 0.26 over 1674 atoms (Figure S12). Nine additional positions proximal to the catalytic residues, or located in the lid domain region, were chosen for site-saturation mutagenesis (M84, F116, G120, R147, G162, F170, K178, P179, and D228) (Figure 3B).

**Figure 3:**
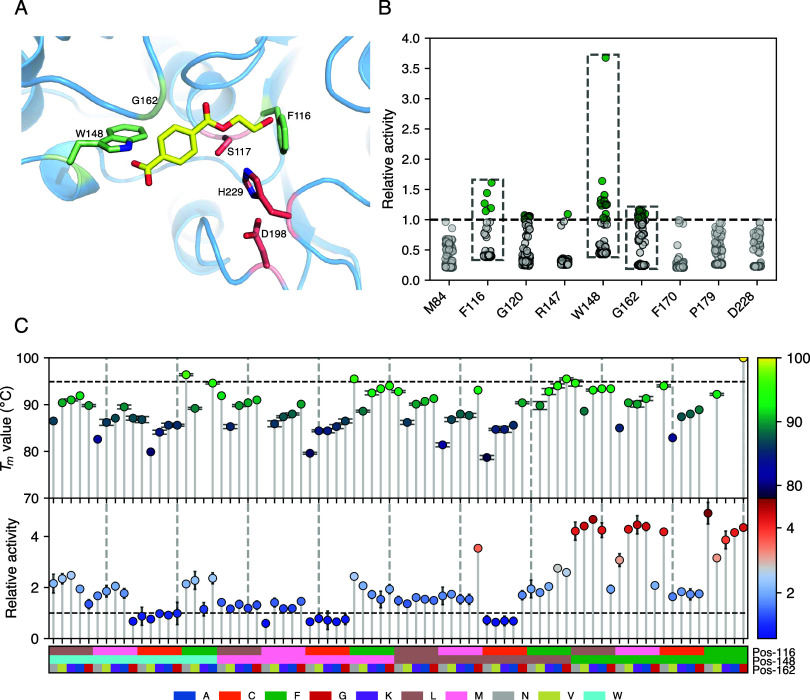
Targeted mutagenesis of the active site and lid domain of MHT077. (A) The top beneficial amino acid residues from the site saturation mutagenesis lysate screen are shown mapped on the crystal structure of MHT077 in green, with MHET (yellow) manually docked in the active site. W148 and F116 flank MHET with F116 proximal to the active site triad (highlighted in pink). G162 is positioned on a loop with the highest B-factors in the structure (Figure S6) and caps the active site region. (B) Jitter plot displaying the relative activity of all variants in the ten single site-saturation mutant libraries of MHT077, with activities normalized as a fold change to the WT control wells of MHT077. Variants were preincubated at 70 °C for 2 h, then reacted with MHET at 70 °C for 1 h, and MHET conversion to TPA quantified by UHPLC. (C) Thermostability and activity screening of purified MHT077 combination variants. Apparent melting temperatures (*T*_m,app_) were determined by differential scanning fluorimetry at a temperature ramp rate of 0.3 °C/s (18 °C/min). End point MHET to TPA % conversion was quantified by UHPLC and is shown normalized as relative activity: fold change relative to WT-MHT077 (dashed horizontal line at 1-fold). Where the thermal unfolding curves displayed maxima above 100 °C, beyond the instrument’s detection range, the *T*_m,app_ of the variant is labeled as not determined (n.d.) in the SI Data set. Activity was assayed at 70 °C, 1 h reaction following a 2 h preincubation at 70 °C. Data are plotted as mean with the error bars displaying the range of two biological replicates for each variant and colored according to the heatmaps shown to the right of each panel. Residue combinations at positions 116, 148, and 162 of the sequence are displayed according to the color map shown below the plot.

Site-saturation mutagenesis libraries for each of these residues were screened as cell lysate in a 96-well plate format. Enzyme variants were first incubated at 70 °C for 2 h to inactivate less thermostable mutants, followed by a MHETase activity assay (2 mM MHET in 0.1 M NaPi pH 8, 1 h, 70 °C) (Figure 3B). All mutations of amino acid residues M84, F170, P179, and D228 were found to be detrimental to activity. However, variants with a positive fold-change in MHETase activity, as normalized relative to the control wells of the wild type (WT) MHT077, were identified within the site-saturation mutagenesis libraries at positions F116, G120, G162, R147, and W148. As hypothesized from our manual docking studies, the variant with the highest MHETase activity score was identified as W148F, with an increase in activity of 3.7-fold.

Next, with the aim of combining potentially additive or synergistic mutations from the single-site saturation screen, all single, double, and triple mutant combinations of the top mutations at F116, W148, and G162 were purified and screened under the same conditions as the previous round (Figure 3C). A double mutant, MHT077^FFN^ and triple mutant, MHT077^LFK^, emerged as the top-performing variants from the screen (see SI Data set for the list of mutations introduced relative to MHT077), exhibiting a 4.7 and 4.9-fold increase in end point MHET conversion to TPA compared to the control, MHT077. Although the MHT077^LFK^ and MHT077^FFN^ variants showed improved activity compared to the single variant at W148F, MHT077^FFG^, the relative fold increase was modest, at 0.3–0.5-fold, respectively. Overall, the W148F substitution, which had exhibited the greatest improvement in the library screen, was present in all the top-performing combinatorial variants (Figure 3C).

### Evolution-Informed Design of MHETase Scaffolds to Achieve Thermostability Improvements

In addition to the targeted active site saturation engineering of MHT077, we also sought to improve several of enzymes identified in the initial HMM search with respect to their *T*_m,app_ and activity. We therefore applied evolution-informed design,^68^ an unsupervised method that proposes highly mutated designs from a parent protein that are consistent with the site-specific and pairwise interaction constraints encoded across sets of homologous sequences. Specifically, 10 *Is*MHETase designs, 20 MHT077 designs, and 10 designs of the other 7 most active MHETases were generated with a target of 10–15% of residues mutated (1–4% for *Is*MHETase). Each design was commercially synthesized and cloned, followed by expression in a 96-well plate format^51^ and characterization of thermostability and activity (Figures 4 and S13–S14).

**Figure 4:**
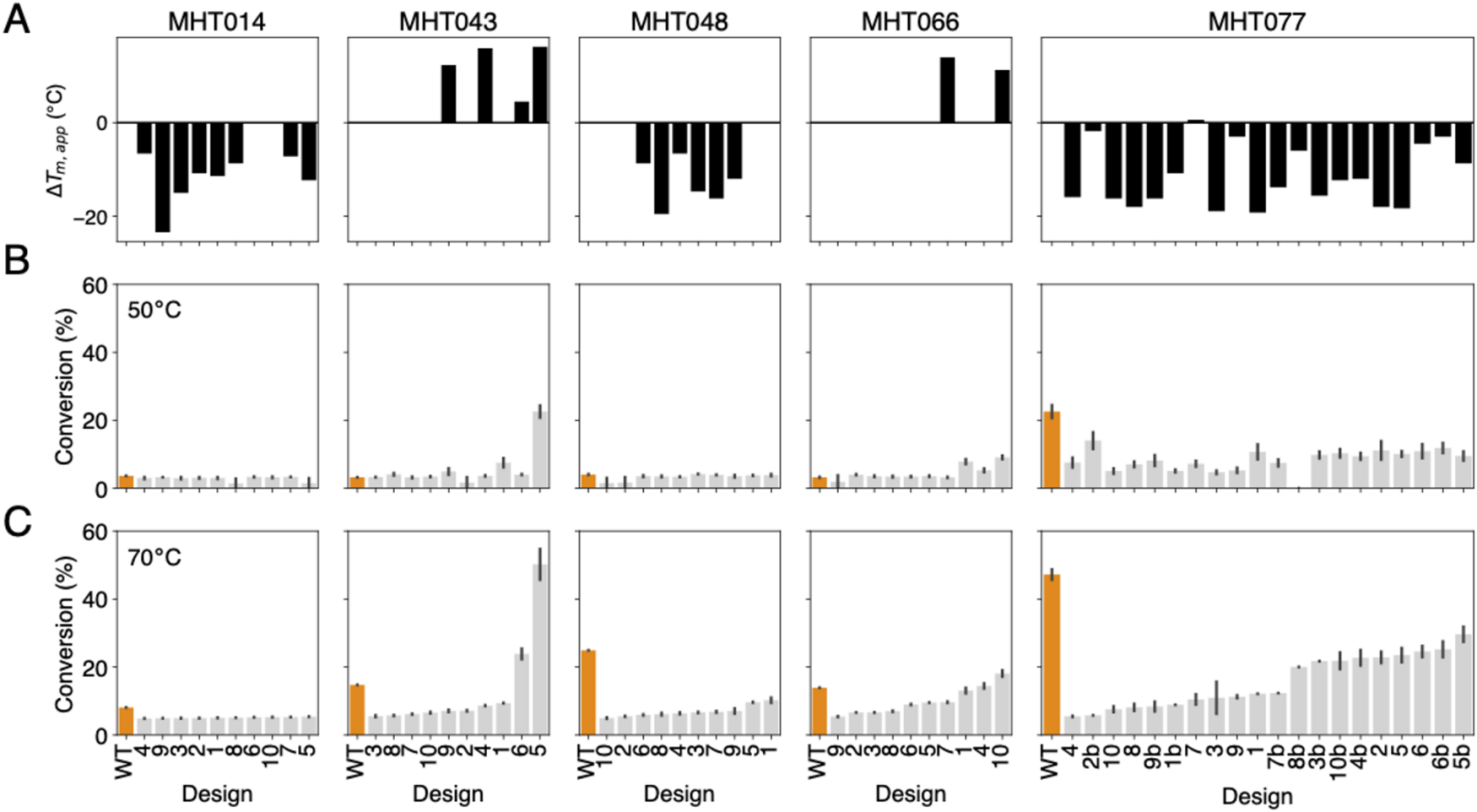
Evolution-informed designs of FAE scaffolds to achieve thermostability improvements. (A) Evolution-informed designs were generated based on MHETase parent sequences (MHT014, MHT043, MHT048, MHT066, and MHT077). Quantification of the change in thermostability (*T*_m,app_) relative to parent sequence. Where no Δ*T*_m,app_ is shown, the melting temperature could not be determined because the variant either lacked a clear unfolding transition or remained folded beyond the 100 °C limit of the assay (see SI Data set for all measured *T*_m,app_ values). (B) MHETase activity of EV-designs at 50 °C (gray bars), expressed as percent MHET conversion. The WT sequence is labeled “WT” and displayed in orange. Bars represent mean values from replicate assays; error bars display the range of two biological replicates. For the MHT077 designs, “b” in the design name denotes the 0.68 design parameters (see Methods in SI). (C) MHETase activity of EV-designs at 70 °C, expressed as percent MHET conversion. Bars represent mean values with error bars centered on the range of two biological replicates.

Improvements in thermostability and activity were achieved in designs based on five of the eight FAEs (Figure 4 and SI Data set). Designs based on MHT022, MHT035, MHT043, MHT045, and MHT066, all showed at least one design with an improvement in thermostability. For activity, improvements in percent MHET conversion were observed for the MHT035, MHT043, and MHT066 designs relative to their respective WT controls (Figure 4b,c and SI Data set). Designs based on *Is*MHETase, MHT014, MHT048, and MHT077 showed no thermostability or activity improvements. Notably, EV-MHT043–5, which contained 36 mutations, showed a 3-fold improvement in MHET conversion relative to WT-MHT043, and a 16.2 °C increase in apparent melting temperature (*T*_m,app_^EV‑MHT043–5^ = 96.1 °C).

Given its activity improved activity at 70 °C and enhanced thermal stability, the crystal structure of EV-MHT043–5 was determined (PDB: 9PJB, Table S1). The mutations that were introduced by the evolution-informed design algorithm relative to the WT-MHT043 are shown in Figure 5. Interestingly, the MHT043 scaffold is functionally resilient to mutation across multiple regions of the enzyme, as mutations were found dispersed across the structure of EV-MHT043–5. A direct comparison of the structure of EV-MHT043–5 overlaid with the MHT077 structure (PDB: 9PIC) is also shown in Figure 5 with the changes highlighted. We observed an increase in similarity to MHT077 of EV-MHT043–5 (bit score 463) compared to the similarity of WT-MHT043 (bit score 433) to MHT077, as determined by BLOSUM62 with equivalent residues according to a Foldmason structural alignment. Of the four mutations introduced by the EVcouplings algorithm within 5 Å of the catalytic histidine, two mutations were to the amino acids observed at the equivalent position in MHT077. Additionally, MHT043 and EV-MHT043–5 both have amino acids F148 and L114 which were observed to be beneficial to MHT077 activity in the mutagenesis portion of this study. Interestingly, by a protein BLAST search,^69^ the WT-MHT043 shares a 100% sequence identity with NCBI GenBank Accession No. RJS94233.1, an α/β hydrolase derived from the *Candidatus* Bathyarchaeota archaeon (sediment metagenome),^70^ the same archaeon from which PET46 was originally discovered.^8^ Accounting for the 36 mutations introduced to the WT-MHT043 sequence by the evolution-informed design algorithm, the EV-MHT043–5 design shares an 86% amino acid sequence identity with RJS94233.1 and a 74% identity with PET46 (RLI42440.1).

**Figure 5:**
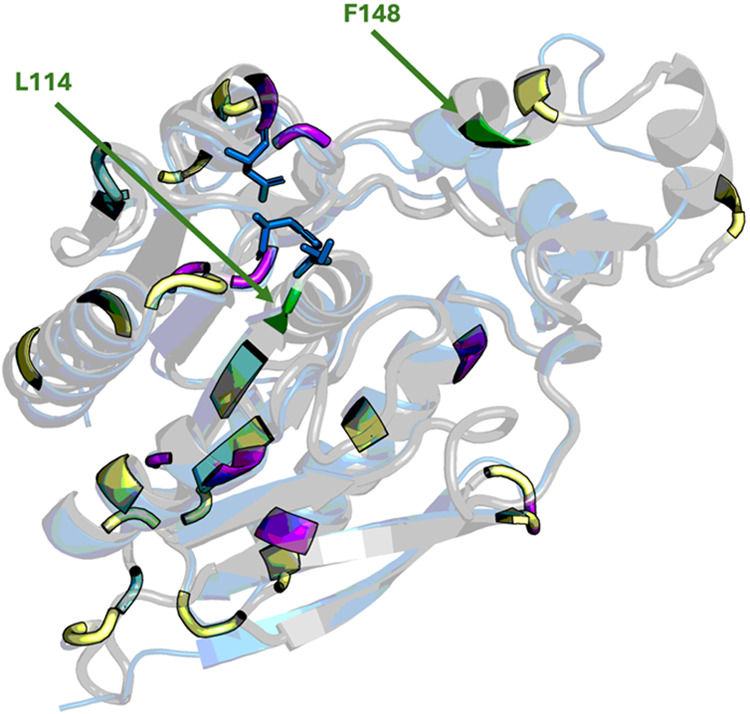
Mutational changes observed in EV-MHT043–5 (PDB: 9PJB, gray) due to the EV couplings algorithm compared to MHT077 (PDB: 9PIC, blue, transparent, active site residues as sticks). The evolution-informed algorithm introduced 36 mutations (outlined, yellow) across the protein relative to WT-MHT043 (RJS94233.1), with 9 mutations to the amino acids observed in MHT077 (purple) and 5 from amino acids in MHT077 (cyan). Two of the 4 mutations made by the algorithm within 5 Å of the catalytic histidine resulted in an amino acid observed on MHT077. Indeed, EV-MHT043–5 is closer to MHT077 (37% identity, bit score 433) than WT MHT043 (35%, bit score 463) is to MHT077 (bit scores according to BLOSUM62). Furthermore, MHT043 and EV-MHT043–5 both have two of the three amino acids (L114, F148) observed to be beneficial when mutated in MHT077 WT (green), with equivalent positions determined using FoldMason^57^ (--refine-iters 5 --report-mode 1 --bitfactor-aa 4.67 --bitfactor-3di 0.387 --gap-open 16). The SI Data set contains the protein sequences of MHT077, WT-MHT043, and EV-MHT043–5.

Ten evolution-informed designs based on *Is*MHETase with an N-terminal deletion were also purified and screened (Figure S15). The N-terminal deletion residues of WT *Is*MHETase were selected by first screening several truncations lengths for expression, as shown in Figure S16. The *Is*MHETase truncations were expressed in pCDB180, a periplasmic secretion vector, due to low expression yields in the pCDB179 vector (data not shown). *Is*MHETase designs were generated with 1–2% (*n* = 5) or 2–4% (*n* = 5) of the length of the sequence mutated (see SI Methods for full design parameters). All of the resulting designs showed decreased *T*_m_ values relative to WT, except EV-*Is*MHETase-1, which retained a *T*_m_ of 51.7 °C (Figure S15). In the activity assay at 50 °C, all designs exhibited lower MHET conversion percentages compared to WT. The structures of the two designs with the highest activities and melt temperatures (EV- *Is*MHETase-1 and EV-*Is*MHETase-5) were predicted by AF3^58^ and are shown in Figure S17. Since no activity was observed for WT at the higher screening temperature of 70 °C (Figure 1a), the *Is*MHETase evolution-informed designs were not evaluated at 70 °C.

### Thermodurability and Kinetic Investigations of Top MHETase Variants

The top-performing MHT077 variants, and EV-MHT043–5, were expressed and purified to investigate their thermodurability under the same MSR-DSC conditions as PET46 and WT-MHT077. The two MHT077 variants exhibited the same native-to-denatured state unfolding behavior as WT-MHT077, as observed in the individual MSR-DSC profiles and the linear Kissinger plots (Figures S18–S19 and Tables S4–S5). At the maximum temperature scan rate employed (192 °C/h), MHT077^FFN^ exhibited a small increase in its apparent *T*_m_ (*T*_m,app_ = 93.6 °C), while MHT077^LFK^ showed a marginal decrease (*T*_m,app_ = 89.7 °C) relative to WT-MHT077 (*T*_m, app_ = 91.7 °C). Overall, the two MHT077 variants exhibited comparable rates of unfolding at 65 °C, with no significant magnitude change in the calculated rates as compared to WT-MHT077 (Tables 1–2). We note that the EV-MHT043–5 design showed lower expression levels at 1-L scale and was therefore not purified in high enough quantity to be included in these analyses. We provide Figure S20 for comparisons of expression titers across the top-performing MHETase variants.

**Table 2:** Calculated Rates of Unfolding Top Variants of MHT077 at 65 °C^e^ ^64^ ^a^,^b^,^c^,^d^

Enzyme	*E*_a_ (kJ/mol)^e^	T* (°C)^e^	*T*_m,app_ (°C)^e^	*k* at 65 °C (s^–1^) (× 10^–8^)^e^
MHT077^LFK^	531 ± 1.3	97.3 ± 0.1	84.0–89.8	7.1 ± 0.4
MHT077^FFN^	501 ± 1.0	102 ± 0.1	87.5–93.6	2.4 ± 0.1

a Energetic analyses of measured DSC thermograms applied an irreversible, native-to-denatured kinetic model using CalFitter v2.0.^64^

b The activation energy for unfolding, where errors indicate the parameter confidences calculated by CalFitter v2.0.

c The temperature at which the kinetic rate constant for the native-to-denatured transition (*k*) is 1 s^–1^.

d The range of apparent *T*_m_ values observed across the scan rate window of 0.2–3.2 °C/min, respectively.

e Calculated from *E*_a_ and T* using the Arrhenius equation (Experimental section).

To compare the activity of the top-performing candidates identified by our two protein engineering strategies, the top variants were assayed against MHET in small-scale reactions (20 mM MHET in 0.1 M NaPi pH 8, 65 °C, Figure S21) (NB: 65 °C was chosen to match the LCC^ICCG^ conditions, *vide infra*). Under these conditions, the MHT077^LFK^ variant exhibited the highest overall conversion. The kinetics of MHT077 and MHT077^LFK^ were also determined at 65 °C. Michaelis–Menten models were fitted to the data using nonlinear regression, and the corresponding *V*_max_ and K_m_ extracted (Figure S22 and Table S6). MHT077^LFK^ exhibited a modest increase in catalytic activity and efficiency (*k*_cat_ = 5.2 ± 0.3 s^–1^) relative to MHT77-WT (*k*_cat_ = 2.5 ± 0.2 s^–1^).

### PET Hydrolysis with a Two-Enzyme System

Subsequently, we evaluated the MHT077^LFK^ variant in combination with a benchmark, thermostable PETase LCC^ICCG^ in pH-controlled bioreactors. Enzymatic PET degradation reactions were performed at the 0.25-L scale at 65 °C using amorphous postconsumer thermoform packaging (PET clamshells) waste of ∼4% crystallinity (see SI Methods, PET characterization, Figure S23, and Table S7). Two conditions were compared: LCC^ICCG^ at an enzyme loading of 0.5 mg/g, and LCC^ICCG^ at 0.5 mg/g in combination with MHT077^LFK^ at 1.5 mg/g added to the reaction after 4 h. We chose LCC^ICCG^ as a benchmark PETase for the two-enzyme system comparison study given that the protein melting temperature of the MHT077^LFK^ variant (*T*_m,app_ = 93 °C) as determined in the Sypro-orange assay, was comparable to LCC^ICCG^ (*T*_m,app_ = 91.7 °C).^14,71^ The initial rates of PET hydrolysis, quantified by NaOH addition, and corresponding MHET and TPA production measured by UHPLC, were established to be consistent across all four reactors over the initial 4 h of the reaction (Figure 6A,B). MHT077^LFK^ addition was carried out at the 4 h time point at the highest measured molar ratio of TPA/MHET (1:0.87). After addition of MHT077^LFK^ to two duplicate reactors at 4 h, a substantial decrease in MHET was measured relative to the control reactors (i.e., only LCC^ICCG^) at the 6 and 8 h time points. Complete final PET conversion after 72 h was determined for both conditions by UHPLC analysis (Figure S24).

**Figure 6:**
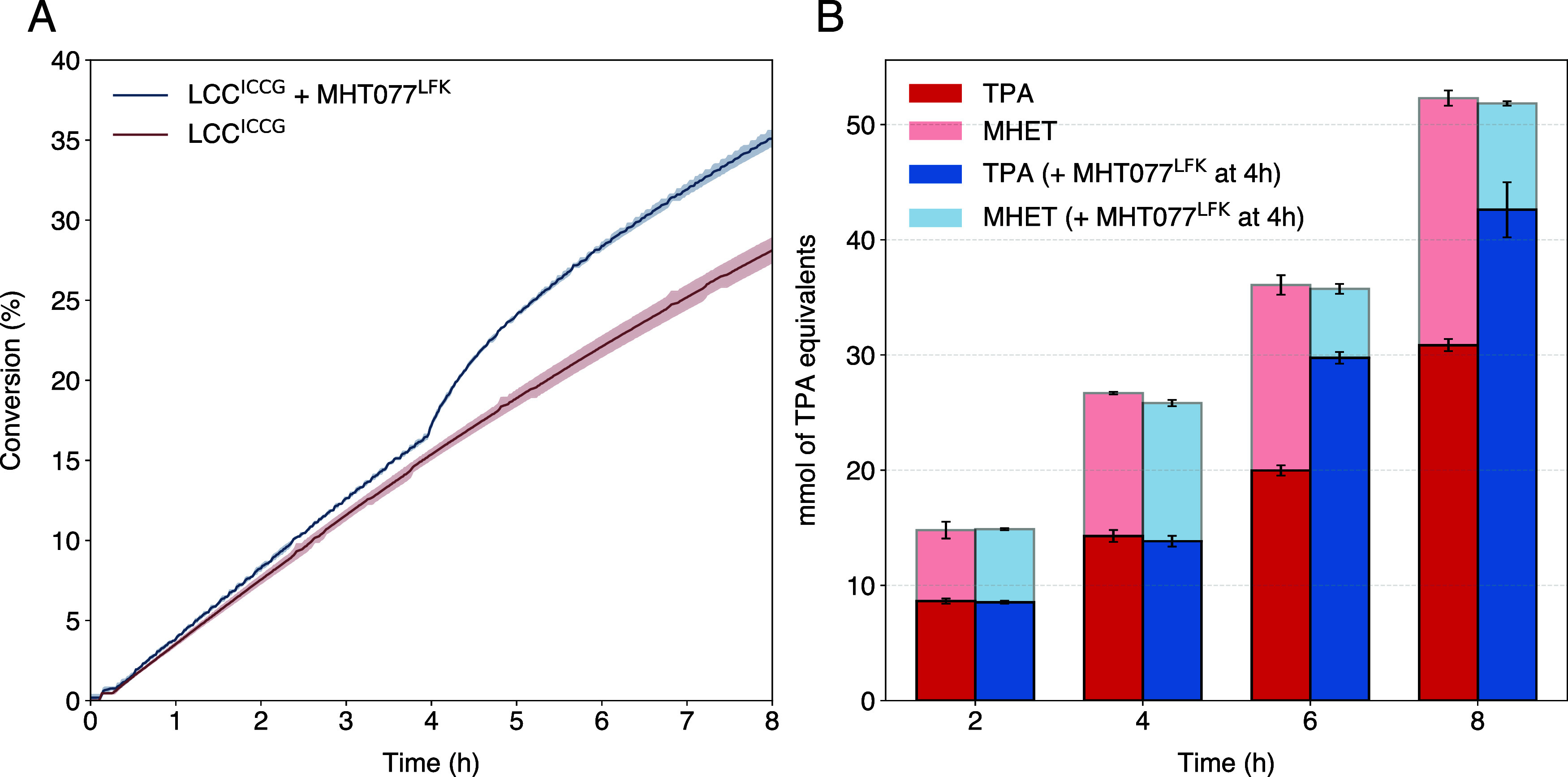
pH-controlled deconstructions of PET with a two-enzyme cocktail of LCC^ICCG^and MHETase variant MHT077^LFK^. (A) Yield analyses of enzymatic deconstruction reactions (15% wt 37.5 g of ground PET thermoforms, 0.25 L, 65 °C) by LCC^ICCG^ (red line, 0.5 mg/g) and LCC^ICCG^ with MHT077^LFK^ (blue line, 0.5 mg/g, 1.5 mg/g MHT077^LFK^ addition at 4 h). The percentage PET conversion is shown calculated by NaOH consumption, where the shaded zones represent the range of two biological replicates. (B) Yields of TPA and MHET over the reaction timecourse as quantified by UHPLC, with error bars centered on the average of two biological replicate reactors. The LCC^ICCG^ condition is shown as red bars with TPA in dark red and MHET in light red, the LCC^ICCG^ with MHT077^LFK^ condition is shown as blue bars with TPA in dark blue and MHET in light blue, as shown in the legend.

## Conclusions

This study provides structural insights into the engineering of lid-domain-covered FAEs, thereby improving both MHET hydrolase activity and thermostability. This campaign therefore lays the groundwork for engineering robust and durable MHETases capable of functioning at the high temperatures required by state-of-the-art PETases. We begin with the identification of scaffolds that display high thermal stabilities, i.e., with melting temperatures above 90 °C. By applying a structure-guided approach with a high-throughput MHET screening strategy, a critical substitution was revealed in the lid domain, with a ∼4-fold improvement in its MHET hydrolase activity with respect to MHT077 at 70 °C. In parallel, we achieved increases in thermostability, up to 16 °C, across five distinct FAEs by application of the EVcouplings method. With the top engineered variant, MHT077^LFK^, we demonstrated the compatibility of thermostable MHETase addition with a benchmark PETase, LCC^ICCG^ and a marked reduction in MHET accumulation in bioreactor deconstructions of postconsumer PET waste. We note that the catalytic efficiency (*k*_cat_/*K*_m_) of MHT077^LFK^ (0.6 mM^–1^·s^–1^) at 65 °C is 6-fold lower than the catalytic efficiency of the KL36F Est30 MHETase at 50 °C (3.64 mM^–1^·s^–1^)^40^ and there remains significant scope for further improvements in the catalytic efficiencies of MHT077^LFK^. While here we pursued a high-temperature screening approach at 70 °C to select for thermostable candidates compatible with thermophilic PETases, we acknowledge that this approach may overlook moderately thermostable but potentially more active candidates.

In this work, we identified enzymes with improved MHET hydrolysis activity based on several FAEs as starting points for enzyme engineering, whereas the *Is*MHETase-based designs that we tested did not exhibit improvements. We anticipate that our strategy of targeting a comparatively smaller lid domain, rather than the ∼240 amino acid lid domain of *Is*MHETase, could be broadly relevant for future enzyme engineering efforts. In particular, a smaller lid domain substantially reduces the number of potential residues that determine MHET specificity, therefore simplifying the design space and enabling more efficient exploration of functionally relevant variants. Future work could target achieving specificity for MHET over PET through surface and lid domain engineering around the active site of MHT077^LFK^. Interestingly, PET46, the FAE upon which we based our original HMM search, was previously reported to degrade semicrystalline PET powder, albeit at low levels compared to benchmark PETases.^8^ Tethering a PETase to a MHETase, for example through a flexible peptide linker has also been shown to enhance the catalytic synergy of the two enzymes in one system.^22,24^ Tethering approaches may help circumvent challenges associated with enzyme occupancy on the PET surface, and therefore potentially overcome surface-crowding effects that limit cooperative activity when the two are applied together in solution. Furthermore, an improved understanding of the evolutionary constraints governing conformational rearrangements of lid domains over serine hydrolase active sites will likely prove central to further activity and specificity enhancements and enable progress toward minimal motif extraction for *de novo* enzyme design efforts. The selective binding and efficient hydrolysis of MHET over PET at high temperatures remains an important objective for future engineering efforts, as achieving specificity will enable co-operativity and chart a course for a more precise enzymatic control over PET processing.

## Experimental Section

### Plasmid Design and Synthesis

All genes used in screening were codon optimized, synthesized, and cloned into the pCDB179 vector (gifted to Addgene by Christopher Bahl, #91960) by Twist Biosciences. *Is*MHETase genes were codon optimized and cloned into the pCDB180 vector (Addgene vector #80677) as constructs with an N-terminal OsmY-10His-Smt3 tag. All pCDB170 constructs consisted of an N-terminal 10xHis tag on the SUMO protein (Smt3) fused to the N-terminus of the target protein to produce tagless proteins as detailed in the purification methods. All codon-optimized DNA sequences and protein sequences are available in the Source Datafile.

### Enzyme Production and Purification

Enzymes were transformed, produced, and purified in a 96-well plate format using the OT-2 robotic protocol previously described.^51^ In brief, two milliliters of Overnight Express Instant TB autoinduction media (Novagen 71491), supplemented with 2× kanamycin and 1× trace metals (Teknova T1001), were dispensed into each well of a 24-deep-well plate (Thomson Instrument Company 931568). Each well was inoculated with 20 μL of a starter culture composed of saturated transformants grown overnight. The inoculated cultures were sealed with breathable AeraSeal adhesive sealing film and incubated at 37 °C for 3 h, followed by growth at 18 °C for two nights at 800 rpm with a 19 mm orbital shaking motion.

Cells were sedimented by centrifugation at 2000*g*, 4 °C, for 10 min and the supernatant discarded. 1.5 mL of Lysis Buffer (20 mM TRIS pH 8.0, 300 mM NaCl, 5 mM imidazole, 1% *n*-octyl β-d-glucopyranoside supplemented with 0.1 mg/mL DNaseI and 1 mg/mL lysozyme) was added to each well. Cells were resuspended by shaking at 18 °C for 2 h. Eight mL of Ni-charged magnetic beads (Genscript L00295) were washed three times with 50 mL Wash Buffer (20 mM Tris pH 8.0, 300 mM NaCl, 5 mM imidazole) and resuspended in 8 mL Wash Buffer. 70 μL of bead suspension was added to each well of a 96-well plate and shaken at 18 °C, 250 rpm for 2 h. After pulling down the beads and discarding the supernatant, the beads were resuspended in 300 μL Wash Buffer, transferred to a new plate, and washed three times with Wash Buffer and twice with 300 μL Cleavage Buffer (20 mM TRIS pH 8.0, 300 mM NaCl). The beads were resuspended in 300 μL Cleavage Buffer, and 30 μL of 0.75 mg/mL His-tagged Cth SUMO protease was added. Cleavage was carried out for 3 h at 1200 rpm (3 mm orbit) at room temperature, then stored overnight at 4 °C and the supernatants transferred to a fresh plate.

### Enzyme Quantification

Concentration was measured using the Pierce Rapid Gold BCA Protein Assay Kit with 10 μL of sample and 200 μL of BCA reagent (50:1 reagent A to reagent B). The assay was incubated at room temperature for 25 min, and the absorbance at 480 nm was recorded. Each plate included calibration BSA solutions prepared in the same buffer as the samples, with concentrations ranging from 0 to 2 mg/mL.

### Dye-Based Protein Thermal Stability Assay

A total of 45 μL from each normalized enzyme well was transferred to a PCR plate (Bio-Rad HSP9601) that contained 5 μL of a 5× solution of Sypro Orange Dye (ThermoFisher Scientific S6650). The plate was sealed (ThermoFisher Scientific 4311971), and fluorescence was measured using a Bio-Rad CFX96 Touch Real-Time PCR instrument with the following program: 1. 25 °C: 0:15; 2. 25 °C: 0:31; 3. 25 °C: 0:15 (+0.3 °C/cycle, temperature ramp rate of 0.3 °C/s (18 °C/min)); 4. Plateread, 5. Go to 3, 250×.

### Multiple Scan Rate Differential Scanning Calorimetry (MSR-DSC)

Enzyme denaturation thermograms were acquired by DSC on a MicroCal PEAQ-DSC Automated instrument (Malvern Panalytical) using the protocol outlined previously.^72^ Immediately prior to DSC analysis, PET46 and MHT077 samples were purified by size exclusion chromatography on a HiLoad Superdex 75 pg column (Cytiva) pre-equilibrated with 50 mM NaH_2_PO_4_/Na_2_HPO_4_, 100 mM NaCl, pH 7.5. For each enzyme, thermograms were recorded in low feedback mode with the temperature raised from 50 to 110 °C at five different ramp rates: 0.2, 0.4, 0.8, 1.6, or 3.2 °C/min. Buffer subtraction and baseline correction were performed using the instrument’s control and analysis software, which also provided the apparent melting temperature (*T*_m,app_). Each enzyme’s thermogram data sets were fit to a single-step, irreversible unfolding model (i.e., native-to-denatured) using the CalFitter v2.0 Web server^64^ which provides *E*_a_, the activation energy for unfolding, and T*, the temperature at which the rate constant (*k*) for the native-to-denatured transition is 1 s^–1^.

At a given temperature (*T*), *k* is related to *E*_a_ and T* by the Arrhenius equation, (eq 1)­

1 $$k=\exp\left(-\frac{E_{a}}{R}\left[\frac{1}{T}-\frac{1}{T{\;}^{*}}\right]\right)$$

where *R* is the universal gas constant (8.314 J/K/mol).

### MHET Hydrolysis Assays

2 mL deep-well 96-well plates were loaded with 418 μL of 100 mM sodium phosphate pH 8 buffer. 50 μL of enzyme (5 μg) was added to each well with the blank containing the same buffer as the enzyme solutions. The plates were sealed Nunc Aluminum Seal Tape (Thermo Scientific 232698) and incubated at the assay temperature (50 or 70 °C) for 2 h with shaking at 800 rpm in a Infors HT Multitron incubator. After 2 h, reactions were initiated by addition of 33 μL of MHET (2 mM final concentration) and the plates incubated for a further 1 h at 50 or 70 °C. After 1 h, the plates were removed, allowed to cool for 45 min, then quenched in an equal volume of methanol prior to quantification of MHET and TPA by UHPLC analysis.

### Amorphous PET Powder Production

Amorphous PET powder was produced by Birch Biosciences from thermoform PET clamshells (Snyder SKU 518685). Label free thermoform PET clamshells supplied by Snyder (100 lbs) were first granulated using a Pagani FAP4090 granulator, with a 0.5-in. screen. This thermoform flake was further sized reduced to generate a powder between 0.5 and 1 mm particle size. Raw flake was placed at −20 °C for 16 h (∼400 g into a plastic bin, 1 in. layer). 100 g of cold flake was transferred to a Homend Electric Grain Mill Grinder (700 model, 36000 r/min) and ground for 10 × 1 s pulses. Post grinder, the material was passed through a 1 mm sieve (18 mesh), removing particles >1 mm, and then passed through a 0.5 mm sieve (35 mesh), removing particles <0.5 mm, resulting in a final powder material with a particle size range of 0.5–1 mm. Percent crystallinity was determined by differential scanning calorimetry (DSC).

### Crystallization, X-ray Data Collection, Structure Determination, and Refinement

#### General

Purified proteins were concentrated to between 10 and 14 mg/mL and each screened across 400–600 crystallization conditions. Crystals were harvested and transferred to a well solution supplemented with 10% glycerol and cryocooled in liquid nitrogen. Diffraction data were collected at the SSRL BL12–2 beamline using a Dectris PILATUS EIGER 2XE 16 M PAD detector. Data were processed with XDS^73^ and solved by molecular replacement using Molrep,^74^ Phaser,^75^ or AmoRE.^76^ The initial refinements were performed through iterative rounds of Refmac^77^ and Coot,^78^ with final rounds using Phenix.^79^ The details of data collection and refinement for all four structures are given in Supporting Table S1.

#### MHT077

The best crystals were obtained from a pH selective screen developed at SSRL (SSRLscreen well G6: 0.075 M KSCN, 0.075 M sodium formate, 0.025 M sodium citrate (pH 5.8), 0.025 M cacodylate (pH 6.5), 0.025 M HEPES (pH 7.4), 0.025 M Tris (pH 8.0), 25% PEG1500). Crystals were grown in sitting drops at 16 °C using a 1:1 ratio of protein to well solution and took around 1 week to grow. The crystals belonged to space group *C*222_1_ with 1 monomer in the asymmetric unit. The structure was solved by molecular replacement using AmoRE by using a pruned model generated from a thermostable lipase (PDB code: 7q4h) as the search model. Refined data extended to a resolution of 1.16 Å.

#### MHT077^W148R^

The crystals used for the data collection were from BCS screen well A10 (0.1 M sodium acetate (pH 4.5), 22% PEG Smear broad). Crystals were grown by sitting drops at 16 °C using a 1:1 ratio of protein to well solution and took around 2 weeks to grow. The crystals belonged to space group *P*2_1_2_1_2_1_ with 2 monomers in the asymmetric unit. The structure was solved by molecular replacement using AmoRE by using the native enzyme as the search model. Refined data extended to a resolution of 1.24 Å.

#### MHT077^E47A^

The best crystals were obtained from a pH selective screen developed at SSRL (SSRLscreen well C4): 25% PEG 3350, 0.15 M K_2_HPO4, 0.02 M citrate (pH 5.8), 0.01 M MES (pH 6.54), 0.02 M Hepes (pH 7.5), 0.02 M ADA (pH 6.5), 0.02 M Tris (pH 8.0). Crystals belonged to space group P2_1_ with 2 monomers in the asymmetric unit. The structure was solved by molecular replacement using AmoRE with the native enzyme as the search model. Refined data extended to a resolution of 1.46 Å.

#### EV-MHT043–5

The best diffracting crystals were obtained from Morpheus screen well H1 (0.1 M amino acids, 0.1 M Buffer system 1, and 30% Precipitant Mix 1). Crystals were grown by sitting drops at 16 °C using a 1:1 ratio of protein to well solution and took around 3 weeks to grow. The crystals belonged to space group *P*2_1_ with 2 monomers in the asymmetric unit. The structure was solved by molecular replacement using Phaser by using the protein model from the MHT077 structure. Initial model building attempts with Buccaneer and Refmac brought the *R*_free_ to around 30%. The structure was further refined by using Refmac and Phenix and manually fitted using the Coot program. Refined data extended to a resolution of 1.77 Å.

### MHT077 Site Saturation Variant Construction

Site saturation mutagenesis libraries were constructed using a standard overlap extension PCR protocol. Degenerate primer pairs were used to introduce the NNK codon at positions identified for mutation, using WT-MHT077 as the gene parent template. Mutant gene libraries were cloned into an empty pET-19b vector by Golden Gate assembly, with variant construction confirmed via Sanger sequencing of the mutant gene inserts.

### Protein Production of Site-Saturation Variants of MHT077

MHT077 variant libraries were expressed in chemically competent *E. coli* C41 (DE3) cells (Lucigen OverExpress). Each well of a sterile microtiter plate containing 150 μL of Luria–Bertani (LB) media supplemented with 100 μg mL^–1^ ampicillin was inoculated with a single colony from a fresh transformation. Plates each contained 6 positive controls of WT-MHT077, and two negative controls of pET19b-RFP (red fluorescent protein). Overnight incubation was then conducted at 30 °C, 80% humidity in a shaking incubator (950 r.p.m.). Expression cultures were prepared by inoculating 480 μL of 2YT media containing 100 μg mL^–1^ ampicillin with 20 μL of overnight culture in deep-well plates, followed by incubation at 30 °C, 80% humidity in a shaking incubator (950 r.p.m.), until an OD_600_ of 1 was achieved. Protein expression was then initiated by the addition of IPTG to a final concentration of 0.1 mM, followed by incubation at 20 °C, 80% humidity in a shaking incubator (950 r.p.m.) for a further 20 h. Cells were collected by centrifugation (2000*g*, 10 min, 4 °C), with cell pellets stored at −80 °C. Cell lysis was initiated by the addition of cell lysis buffer (20 mM Tris pH 8.0, 300 mM NaCl, 5 mM imidazole, 1% *n*-octyl β-d-glucopyranoside supplemented with 0.1 mg/mL DNaseI and 1 mg/mL lysozyme), followed by incubation for 2 h at 20 °C, 80% humidity in a shaking incubator (950 r.p.m.). The resulting cell lysate was clarified by centrifugation (2,000*g*, 20 min, 4 °C) and stored at 4 °C prior to MHETase assay screening.

### Screening of Site-Saturation Variants of MHT077

For screening, 50 μL of the clarified lysate was transferred to 96-well microtiter plates containing 417 μL of reaction buffer (100 mM sodium phosphate pH 8). Reactions were initiated by addition of MHET in phosphate buffer pH 8 at a final reaction concentration of 2 mM. Formation of the TPA reaction product was monitored by UHPLC. The most active variants for each site-saturation mutagenesis library were rescreened in triplicate.

### Construction of Combinatorial Variants of MHT077

Beneficial diversity identified in the site-saturation mutagenesis library screening was combined to produce double and triple mutants. Mutant genes were ordered as pCDB179 plasmids from Twist Biosciences (see section on plasmid design).

### pH Activity Screen of MHT077 and PET46

Dilutions of the purified protein, MHT077 or PET47, (5 μg final) were prepared in a Britton-Robinson buffer in single pH unit intervals across pH 2–9. For pH 8, 100 mM sodium phosphate pH 8.0 was included as an additional standardized buffer control. All samples were incubated for 1 h at 70 °C and product release quantified by UPLC.

### Evolution-Informed Design

We applied evolution informed design to generate sequence variants of IsMHETase (*n* = 10) and the and the 8 HMM-identified enzymes (*n* = 20 for MHT077 and *n* = 10 for the rest):

1. A multiple sequence alignment (MSA) of homologous enzymes was derived for each starting chassis using the jackhmmer tool.^80^
2. EVcouplings models, which account for positional constraints and pairwise interactions, were then parametrized using each of these MSAs.^81,82^ We modeled a variety of MSA depths (specified by bitscore) for each chassis, and selected the top MSA that (1) maximized sequence coverage, (2) had a number of effective sequences over the length (Neff/L) of approximately 10, and (3) had reasonable overlap of model interactions with known structural contacts. For MHT077, we also generated an additional 10 sequences (20 total) from a model trained on a larger alignment (Neff/L of ∼43).
3. The predicted fitness of arbitrary sequences are then defined as the evolutionary Hamiltonian (EVH), which is applied during a sampling procedure (Gibbs Sampling) procedure to design variants with an objective function that aims to (1) maximize the predicted fitness (EVH), (2) ensure a specified number of mutations (e.g., 10–15% of the primary sequence), and (3) enforce diversity between each design and natural sequences in the MSA.
4. For *is*MHETase we generated 10 designs on a chassis that consisted of an N-terminal deletion variant (length = 568 amino acids), in which the first 35 residues were removed. This variant was successfully expressed and remained functional (Figure S15). Gibbs sampling was applied twice to generate 10 total IsMHETase designs (*n* = 5 for each parameter set): ParamSet1 aimed for 1–2% mutations in the primary sequence and a minimum of 3% sequence difference between designs (h99), while the second aimed for 2–4% of the sequence to be mutated with a minimum of 4% difference between designs (h98). Applying these parameter sets resulted in 9–12 [9,9,9,11,12] mutations and 17–18 [∼17,17,17,17,18] mutations for ParamSet1 and ParamSet2, respectively.
5. For designs based on the 8 HMM-identified enzymes, 10 designs were generated with parameters aiming for 10–15% mutations in the primary sequence and a minimum of 10% sequence differences between designs.

### UHPLC Analysis

TPA, MHET, and BHET were analyzed by ultrahigh performance liquid chromatography (UHPLC) as previously detailed.^83^ Briefly, samples and standards were analyzed by UHPLC coupled with diode array detection (DAD). Chromatographic separation was accomplished using a mobile gradient consisting of 20 mM phosphoric acid and methanol and a Zorbax Eclipse Plus C18 Rapid Resolution HD analytical column. A quantification wavelength of 240 nm was used to construct calibration curves for quantitation of each analyte of interest.

### Kinetic Measurements of MHET Hydrolysis

Enzyme reactions were performed in duplicate over 35 min using purified enzymes (0.3 μM) and MHET substrate (2–20 mM) in 0.1 M sodium phosphate pH 8 at 65 °C. Reactions were quenched in an equal volume of precooled methanol (500 μL) prior to UHPLC analysis of MHET and TPA. Initial reaction velocities were determined by linear regression of the time course data, and kinetic parameters were obtained by nonlinear regression fitting to the Michaelis–Menten equation.

### Differential Scanning Calorimetry of PET Thermoforms

Differential scanning calorimetry (DSC) measurements were conducted in triplicate measurements using a Discovery DSC 25 (TA Instruments). The reported data represents the average of triplicate measurements, and all data was obtained from the specified first heating and cooling cycle. Samples of 2–3 mg of the PET Grind material were placed in hermetic aluminum pans (TA Instruments–PN# 901683.901) and sealed with hermetic pinhole lids (TA Instruments–PN# 901684.901). The pinhole was created using a small needle and plastic hammer. Analysis involved equilibrating samples at 0 °C. The samples were then heated at 10 °C/min to 300 °C, held at 300 °C for 5 min, and cooled at 10 °C/min to 0 °C. The glass transition temperature (*T*_g_) was determined from the midpoint of transition. The melting point was determined from the peak temperature of melting transition. The enthalpy of melting used for 100% crystalline PET was 140.1 (J/g). The glass-transition temperature (*T*_g_), heat of melting (Δ*H*_m_), and heat of cold crystallization (Δ*H*_cc_) were determined with TA Instruments Trios software.

Percent crystallinity was calculated using eq 2, where Δ*H*_m_° is the reference heat of melting for PET = 140.1 J g^–1^.


2 $$\%\;\mathrm{crystallinity}=\left[H_{m}-H_{\mathrm{cc}}\right]/{H_{m}}^{{}^{\circ}}\times 100\%$$

### Bioreactor Reactions with a Two-Enzyme System

Bioreactor reactions were initiated at 0.25-L scale in duplicate 1-L glass bioreactors (Applikon Biotechnology), which included two Rushton impellers in the stirrer shaft below the 0.2-L line. The PET substrate used was ground clamshells (supplied by Birch Biosciences). A total of 37.5 g of substrate (15 wt %) was added to 100 mM sodium phosphate pH 8 assay buffer at a final volume of 0.25 L and equilibrated to 65 °C with stirring at 300 rpm. All reactors were initiated by the addition of 3.4 mL of 5.5 mg mL^–1^ of LCC^ICCG^ for a final enzyme loading of 0.5 mg per g of PET and proceeded for 72 h maintained at pH 8 with 4 M NaOH addition using a peristaltic pump controlled by an in-control module (Applikon Biotechnology). After 4 h, 10 mL of 5.6 mg mL^–1^ of MHT077^LFK^ was added to two reactors in duplicate. Samples were collected at 2, 4, 6, 8, 24, 48, and 72 h and stored at 4 °C prior to UPLC analysis of TPA, MHET and BHET analytes.

## Supplementary Material





## Notes

Additional experimental and computational characterization, methods, structural similarity tree of putative MHETases identified in the phmmer search; taxonomy and genomic context of putative MHETases; structural diversity of MHETase lid domains; comparison of lid domain mobility; crystallographic parameters, data collection and refinement statistics; MSR-DSC analyses of MHT077, and MSR-DSC analyses of PET46 (PDF)
Apparent melting temperature and activity data for all enzymes and the source data for Figures 1, 3, 4, and 6 (XLSX)
