# Supplementary material for: Engineering and Application of a Thermostable MHETase for PET Depolymerization
Source: ACS Sustain Chem Eng. 2026 Jun 8;14(24):10981–95. doi: 10.1021/acssuschemeng.6c01404 (PMC13292399; doi:10.1021/acssuschemeng.6c01404)
Supplement: Supplementary file 1 [file sc6c01404_si_001.pdf]

Supporting Information

Engineering and application of a thermostable MHETase for PET depolymerization

Natasha P. Murphy,[1,2,3] Japheth E. Gado,[1,2,3] Tabea Neumann,[4] Pablo Perez-Garcia,[4] Evan Komp,[1,3] Luisana Avilan,[5] Irimpan I. Mathews,[6] Elizabeth L. Bell,[1,2] Brenna Norton-Baker,[1,2,3] Matilda Clark,[5] Rebecca R. Garcia,[1] Hannah M. Alt,[1] Ritimukta Sarangi,[2,6] Andrew R. Pickford,[5] Wolfgang R. Streit,[4] John E. McGeehan,[1,2] Nicholas P. Gauthier,[7,8] Gregg T. Beckham[1,2,3,*]

1. Renewable Resources and Enabling Sciences Center, National Laboratory of the Rockies, Golden, CO 80401, USA
2. BOTTLE Consortium, Golden, CO 80401, USA
3. Agile BioFoundry, Emeryville, CA 94608, USA
4. Department of Microbiology and Biotechnology, University of Hamburg, Hamburg, 22609, Germany
5. Centre for Enzyme Innovation, School of the Environment and Life Sciences, University of Portsmouth, Portsmouth, PO1 2DY, UK
6. SLAC National Accelerator Laboratory, Stanford Synchrotron Radiation Lightsource, Menlo Park, CA 94025, USA
7. Department of Systems Biology, Harvard Medical School, Boston, MA 02115, USA
8. Department of Data Sciences, Dana-Farber Cancer Institute, Boston, MA 02115, USA

* correspondence: gregg.beckham@nlr.gov

Number of pages: 25

Number of figures: 24

Number of tables: 7

Table of Contents

Supporting Information 1

Hidden Markov model search and screen *5*

Hierarchical tree of structural similarity of HMM and literature MHETases *5*

Figure S1. Structural similarity tree of putative MHETases identified in the phmmer search. Structures for each candidate were predicted with AF3 and used to create a structural alignment with Foldmason (default parameters with 100 refinement iterations). Output pairwise LDDTs were renormalized to the average sequence length of the pair of sequences considering gaps as 0.0. The negative natural logarithm of these LDDTs were used to compute the tree branches according to UPGMA.[1,2] All the MHT sequences screened in this study can be found in the Source Datafile.......... 5

Figure S2. Experimentally measured MHETase activity of all HMM candidates, shown as percent conversion at (A) 50 ºC and (B) 60 ºC, plotted against their respective thermal stability, with *Is*MHETase (pink) and MHT077 (dark blue) highlighted. Apparent melting temperatures were determined by the Sypro-orange dye-binding assay at a temperature ramp rate of 0.3 °C/s (18 °C/min). Error bars represent the range in activity between biological duplicates. If two inflection points were observed, the highest was used for the measurable T_m. The dashed line indicates the autohydrolysis conversion of the MHET substrate control to TPA in 100 mM phosphate pH 8.......... 6

Figure S3. Taxonomy and genomic context of putative MHETases. PET46, *Is*MHETase, and 87 MHT sequences. Active sequences in this study are highlighted in bold; *Is*MHETase, PET46, and MHT077 are displayed in the same colors as in Figure 1. Sequences were aligned using Clustal Omega.......... 7

Structural comparisons of HMM MHETase candidates *8*

Figure S4. Electrostatic distribution. Electrostatic potential distribution mapped to the solvent-accessible surface of PET46 (a, c) and MHT077 (b, d), colored as a gradient from red (acidic) at −7 kT/e to blue (basic) at 7 kT/e (where k is the Boltzmann's constant, T is temperature, and e is the charge of an electron).The narrow substrate binding pocket is highlighted with a black oval in each enzyme. Rotation by 90 degrees around the horizontal axis (c and d) reveals a highly acidic patch (red) on PET46 that is absent in MHT077 (while, neutral). Both enzymes have a corresponding basic pocket (blue).......... 8

Figure S5. Structural diversity of MHETase lid domains. Aligned lid domains are ordered based on increasing RMSD from PET46. Flanking models correspond to X-ray structures for PET46 (PDB 8B4U) and MHT077 (PDB 9PIC), and the intervening models were generated with AlphaFold.......... 9

Figure S6. Comparison of lid domain mobility. Lid domains are rendered for (a) PET46 (PDB 8B4U) and (b) MHT077 (PDB 9PIC) and colored by B-factor scaled to the overall structure, including the core domain (not shown). The reduction in secondary structural elements for the MHT077 lid domain correlates with high B-factors and may indicated higher dynamic mobility of this region.......... 9

Table S1: Crystallographic parameters, data collection and refinement statistics.......... 10

Table S2. MSR-DSC analyses of MHT077.......... 12

Table S3. MSR-DSC analyses of PET46.......... 12

Figure S7: Enzyme $T_{m.app}$ values from MSR-DSC thermograms are dependent on temperature scan rate. DSC thermograms for (a) PET46, and (b) MHT077 recorded with the indicated temperature scan rates. The profiles are consistent with a native-to-denatured model and an irreversible unfolding mechanism. Total area shaded under curves are shown in Tables S2 and 3. Each $T_{m,app}$ is shown inset.......... 12

Figure S8: Linear Kissinger plots of VSR-DSC fits for MHT077 and PET46, consistent with an irreversible, native-to-denatured kinetic model.......... 13

Figure S9: Activity of PET46 (a) and MHT077 (b) at single unit pH intervals across the range of pH 2-8 (1 hour reaction, 65 °C). The Britton-Robinson buffer was used to prepare each reaction condition. For comparison purposes a standard 100 mM sodium phosphate pH 8 condition was included.......... 13

Structure-based site-saturation engineering of MHT077 .... *14*

Figure S11. Comparison with homologous crystal structures. (a) Superposition of MHT077 in blue with docked MHET in yellow, and cinnamoyl esterase (3S2Z)[9] in light orange with bound caffeic acid in magenta. Equivalent active site residues in each structure are shown as sticks. (b) Close up comparison of the active site regions demonstrates that docking MHET onto the aromatic portion of caffeic acid results in a steric clash with MHT077 residue Trp148. (c) Superposition of MHT077 (blue) to ferulic acid esterase (7Z2U)[10] in green, with

bound ferulate also in green. (d) The bound ferulate ligand of ferulic acid esterase adopts a similar position to caffeic acid in cinnamoyl esterase, also sterically incompatible with docking MHET in proximity to Trp148. 15
Figure S12. Comparison of parent MHT077 and MHT077^{W148R}. A superposition of coordinates from MHT077 (9PIC, blue) and MHT077^{W148R} (9PID, pink) show no significant change in the overall structure beyond the W148R substitution. The active site triad residues in each structure are also rendered as sticks for reference.. 15

Evolution-informed design of MHETase candidates *16*
Figure S13. Model quality of *Is*MHETase determined by comparison of the overlap of predicted residue-residue interactions (”couplings”) and structural contacts available in PDB structures. (A) *Is*MHETase EVcouplings model derived from a functional n-terminal truncation variant lacking the first 35 amino acids (bitscore parameter, which determines the number of homologs used for modeling was set to 0.6). The top L (length of sequence) predicted residue-residue interactions for each that are at least 5 positions apart in primary sequence are displayed (blue and red dots) and used for percentage calculations below each plot. Structural contacts were determined by the EVcouplings compare tool.[11] Dark blue dot: model-predicted interaction that is also a PDB structural contact. Red dot: model-predicted interaction that is not a PDB structural contact. Gray dots: PDB structural contact defined as residues within 5 angstroms of one another (any atom). Red bar: Positions that are not considered in the model, which indicate many gaps in the multiple sequence alignment used for model inference. 16
Figure S14. Model quality of natural MHETase scaffolds determined by comparison of the overlap of predicted residue-residue interactions (”couplings”) and structural contacts available in PDB structures. The identity of each natural MHETase is indicated above the plot as well as the bitscore (an indicator of how many homologs are used for model inference). The top L (length of sequence) predicted residue-residue interactions that are at least 5 positions apart in primary sequence are displayed (blue and red dots) and used for percentage calculations below each plot. Structural contacts were determined by the EVcouplings compare tool.[11] Dark blue dot: model-predicted interaction that is also a PDB, structural contact. Red dot: model-predicted interaction that is not a PDB structural contact. Gray dots: PDB structural contact defined as residues within 5 angstroms of one another (any atom). Red bar: Positions that are not considered in the model, which indicate many gaps in the multiple sequence alignment used for model inference. 17
Figure S15: (a) Apparent protein melting temperatures determined by the Sypro-orange dye-binding assay at a temperature ramp rate of 0.3 °C/s (18 °C/min) and (b) MHET hydrolase activity at 50 ºC of 10 EV designs based on *Is*MHETase (WT control shown in orange). Error bars represent the range between biological duplicates. The two sets of EV couplings parameters that were applied to generate the designs (denoted by h98 and h99 respectively) are described in the Methods. 18
Figure S16. SDS PAGE gel of purified *Is*MHETase proteins with N-terminal truncations. The *Is*MHETase truncations were expressed in pCDB180. Samples of purified *Is*MHETase N-terminal deletion proteins, Δ15, Δ16, Δ17, Δ35, and Δ45, with the supernatants from expression of Δ16-45 shown tor the right alongside marker (lane 1 – molecular weights denoted). Samples were boiled in Laemmli buffer at 95 °C prior to loading on the gel. The proteins were only observed in the lysates samples. 18
Figure S17: Overlayed AlphaFold3 predicted structures of EV-design-h99-1 and EV-design-h99-5 based on *Is*MHETase. The 9 mutations introduced by the EVcouplings method into design 1 are shown as pink spheres, and the 12 mutations of design 5 shown as blue spheres. Overlapping mutation sites are shown as split-colored blue and pink spheres. The SI Dataset contains the individual protein sequences. 19
Figure S18: Enzyme kinetic stability analysis from MSR-DSC. DSC thermograms for (a) MHT077^{LFK} and (b) MHT077^{FFN}, recorded with the indicated temperature scan rates. The profiles are consistent with a native to denatured model and an irreversible unfolding mechanism. Total area shaded under curves are shown in Tables S4-5. Each $T_{m,app}$ value is shown inset. 20
Table S4. Kinetic stability analyses from MSR-DSC thermograms of MHT077^{LFK}. 21
Table S5. Kinetic stability analyses from MSR-DSC thermograms of MHT077^{FFN}. 21
Figure S19. Linear Kissinger plots of MSR-DSC fits for MHT077 variants, MHT077^{LFK} and MHT077^{FFN}, consistent with an irreversible, native-to-denatured kinetic model. 21
Figure S20. Scatter plot showing expression titers of MHETase variants with the top-performing MHETase variants, MHT077-WT, MHT077-LFK, MHT077-FFN and EV-MHT043-5 marked as blue crosses. Expression values are plotted in mg/mL, where all variants were expressed as described in the Enzyme production and purification section of the Experimental Methods 22
Figure S21. MHETase activity of PET46, MHT077-WT, MHT077 variants, and EV-MHT043 at 65 ºC in small-scale reactions (20 mM MHET in 0.1 M NaPi pH 8, 65 ºC), expressed as percent MHET conversion.

Bars represent mean values with error bars centered on the range of two biological replicates. An autohydrolysis substrate control of 20 mM MHET in 100 mM sodium phosphate pH 8 is shown labelled as 'MHET_control'. 22
Figure S22. Kinetics of MHT077 WT and MHT077^{LFK} measured at 65 ºC in 0.1 M sodium phosphate pH 8. Initial rates of TPA production, calculated using linear regression, are shown plotted against MHET concentration (0-20 mM). A Michaelis-Menten kinetics model was applied to fit each curve and extract the k_{cat} and K_m values shown in Table S6. Error bars represent mean values with error bars centered on the range of two replicates. 23
Table S6. Kinetic parameters of WT-MHT077 and the MHT077^{LFK} variant at 65 ºC. 23
Figure S23. Differential scanning calorimetry (DSC) thermograms of ground thermoform PET substrate. Exothermic is up, endothermic is down. Percent crystallinity was calculated via Equation S1 (see Methods) and is presented in **Table S7**........ 23
Table S7: Thermal properties and crystallinity of the three replicates of amorphous ground thermoform PET shown in **Figure S23**........ 23
Figure S24: Yields of pH-controlled deconstructions of PET (37.5 g in 0.25 L) with LCC^{ICCG} (red) and a two-enzyme cascade of LCC^{ICCG} and MHETase variant, MHT77^{LFK} (blue). TPA and MHET analytes were determined by UHPLC. Error bars are centered on the average of two duplicate reactors. 24

Hidden Markov model search and screen

Hierarchical tree of structural similarity of HMM and literature MHETases

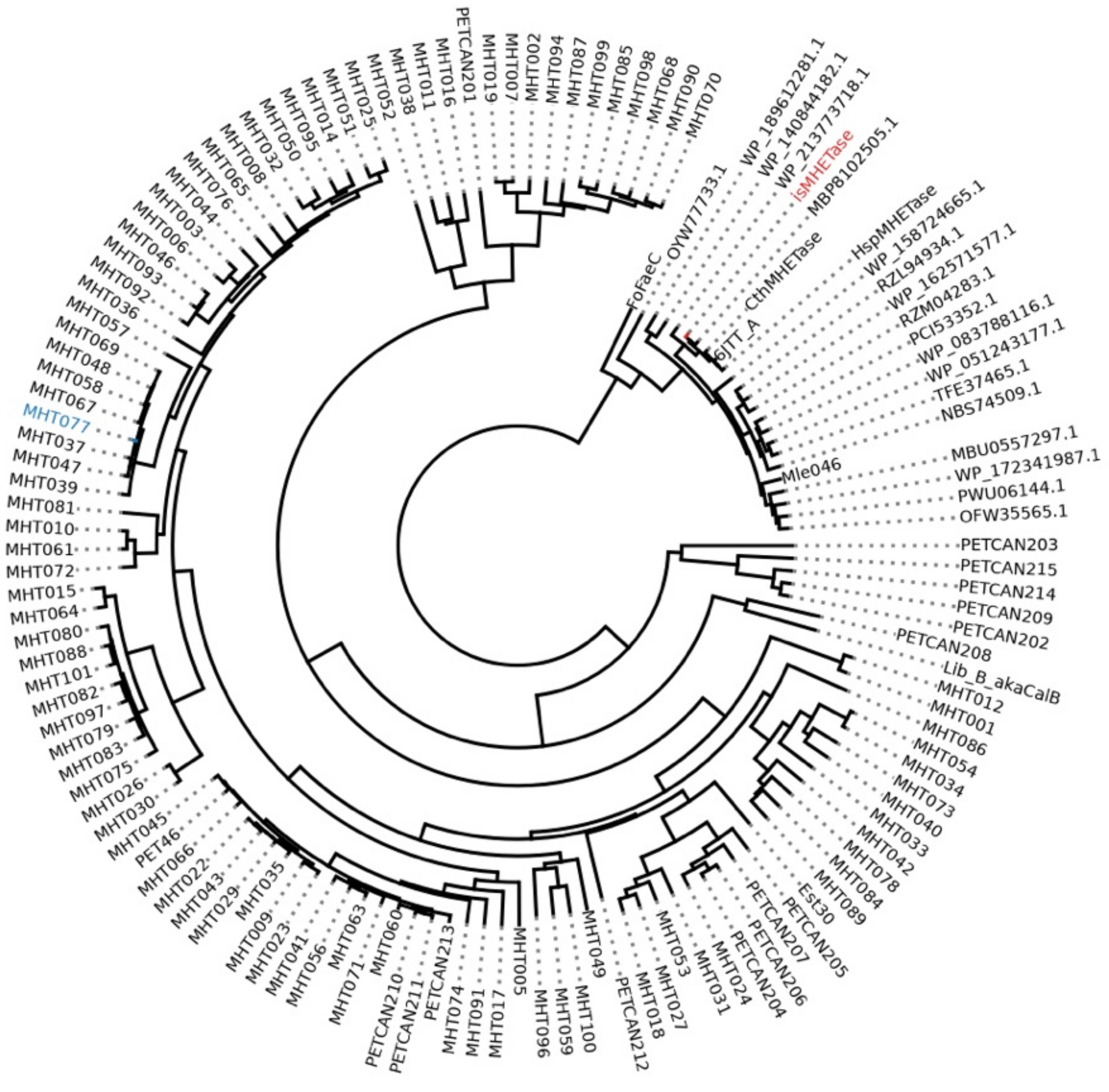

Figure S1. Structural similarity tree of putative MHETases identified in the phmmer search. Structures for each candidate were predicted with AF3 and used to create a structural alignment with Foldmason (default parameters with 100 refinement iterations). Output pairwise LDDTs were renormalized to the average sequence length of the pair of sequences considering gaps as 0.0. The negative natural logarithm of these LDDTs were used to compute the tree branches according to UPGMA.[1,2] All the MHT sequences screened in this study can be found in the Source Datafile.

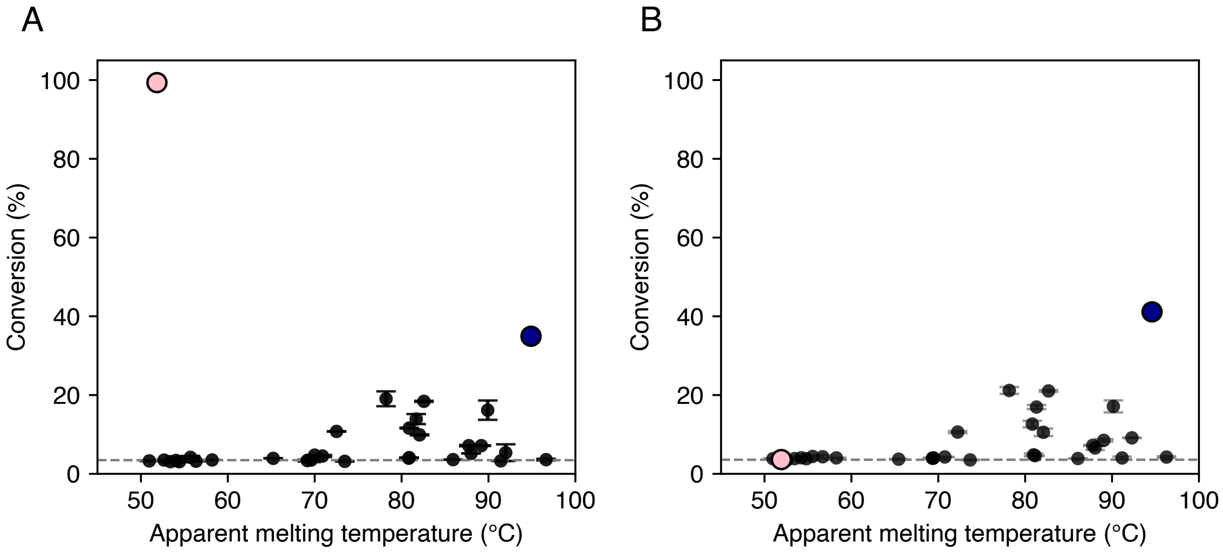

Figure S2. Experimentally measured MHETase activity of all HMM candidates, shown as percent conversion at (A) 50 ºC and (B) 60 ºC, plotted against their respective thermal stability, with *Is*MHETase (pink) and MHT077 (dark blue) highlighted. Apparent melting temperatures were determined by the Sypro-orange dye-binding assay at a temperature ramp rate of 0.3 °C/s (18 °C/min). Error bars represent the range in activity between biological duplicates. If two inflection points were observed, the highest was used for the measurable T_m. The dashed line indicates the autohydrolysis conversion of the MHET substrate control to TPA in 100 mM phosphate pH 8.

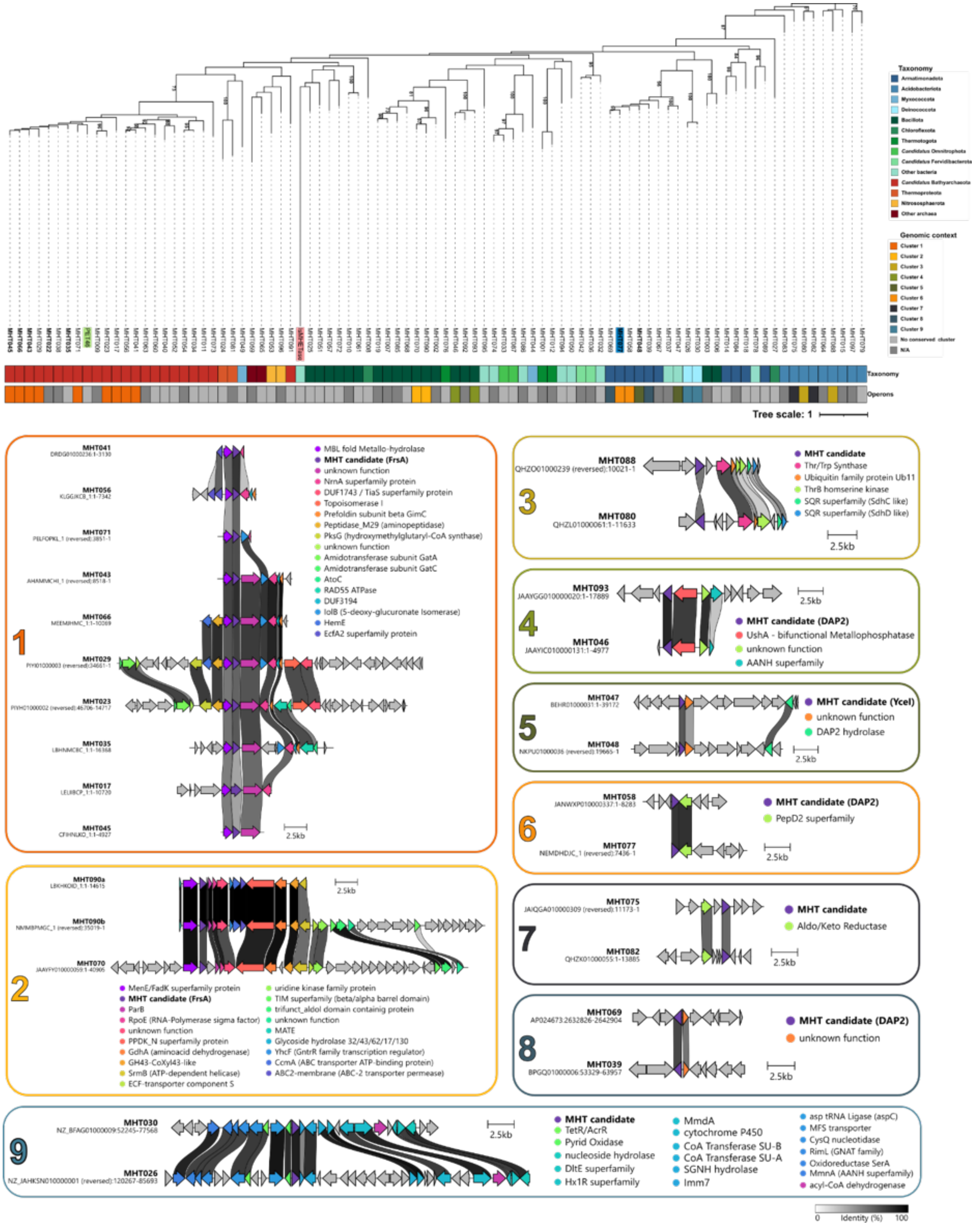

Figure S3. Taxonomy and genomic context of putative MHETases. PET46, *Is*MHETase, and 87 MHT sequences. Active sequences in this study are highlighted in bold; *Is*MHETase, PET46, and MHT077 are displayed in the same colors as in Figure 1. Sequences were aligned using Clustal Omega.[3] Phylogenetic analysis was performed with RAxML-NG,[4] employing the LG substitution matrix, 8 discrete GAMMA categories, and 10 randomized parsimony starting trees. Node support was assessed using 200 bootstrap

replicates; only values ≥70% are shown. Taxonomy was assigned based on the best hit from a DIAMOND v.2.0.15[5] blastp search against the NCBI nr database. For each identified MHT homolog, the corresponding full-length contigs or scaffolds were retrieved from both NCBI GenBank and the MGnify database using their respective API endpoints. MGnify-recovered contig sequences were annotated using Prokka v1.14.6[6] with default parameters for prokaryotic genomes. Annotated genomic regions containing MHT homologs were then analyzed with Clinker v0.0.31[7] to identify conserved genes and operon-like structures. To further characterize predicted gene products, particularly those lacking clear annotations, all protein sequences were analyzed with CD-Search[8] for conserved domains and functional prediction of hypothetical open reading frames in MHT gene neighborhoods.

Structural comparisons of HMM MHETase candidates

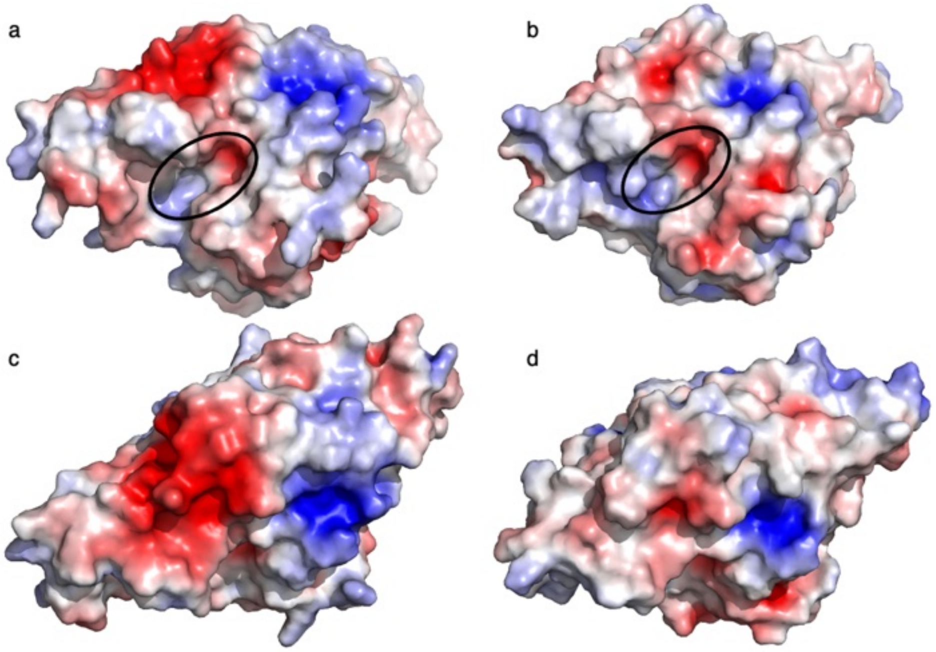

Figure S4. Electrostatic distribution. Electrostatic potential distribution mapped to the solvent-accessible surface of PET46 (a, c) and MHT077 (b, d), colored as a gradient from red (acidic) at −7 kT/e to blue (basic) at 7 kT/e (where k is the Boltzmann's constant, T is temperature, and e is the charge of an electron).The narrow substrate binding pocket is highlighted with a black oval in each enzyme. Rotation by 90 degrees around the horizontal axis (c and d) reveals a highly acidic patch (red) on PET46 that is absent in MHT077 (while, neutral). Both enzymes have a corresponding basic pocket (blue).

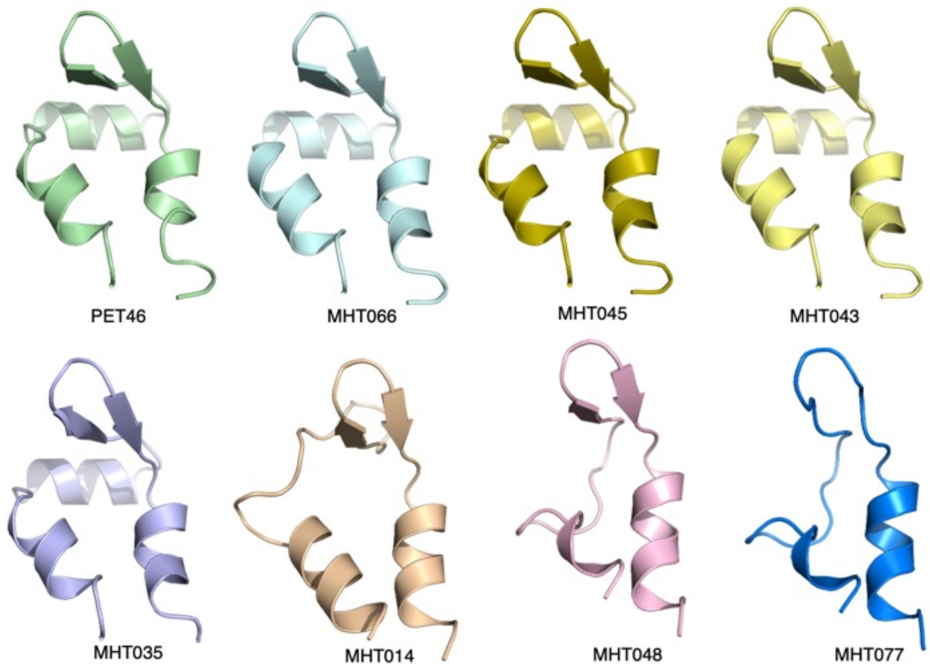

Figure S5. Structural diversity of MHETase lid domains. Aligned lid domains are ordered based on increasing RMSD from PET46. Flanking models correspond to X-ray structures for PET46 (PDB 8B4U) and MHT077 (PDB 9PIC), and the intervening models were generated with AlphaFold.

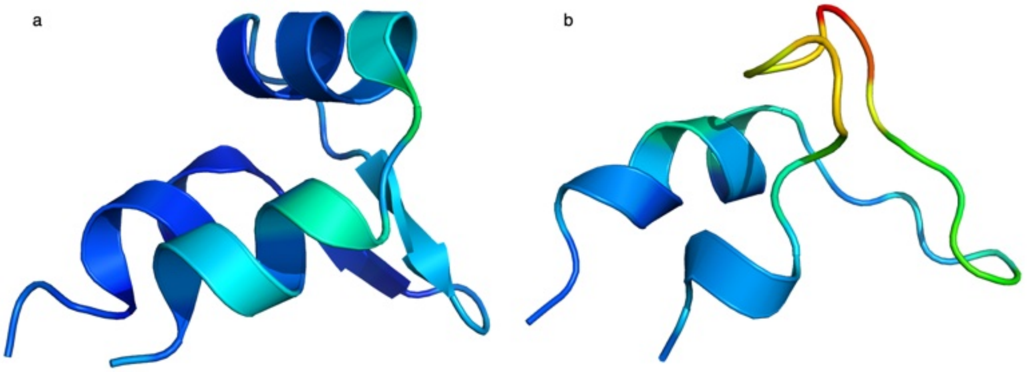

Figure S6. Comparison of lid domain mobility. Lid domains are rendered for (a) PET46 (PDB 8B4U) and (b) MHT077 (PDB 9PIC) and colored by B-factor scaled to the overall structure, including the core domain (not shown). The reduction in secondary structural elements for the MHT077 lid domain correlates with high B-factors and may indicated higher dynamic mobility of this region.

Table S1: Crystallographic parameters, data collection and refinement statistics.

	MHT077 (PDB: 9PIC)	$MHT077^{W148R}$ (PDB: 9PID)
**Crystallographic parameters**		
Space group	$C222_1$	$P2_12_12_1$
Unit-cell dimensions	47.84, 109.11, 92.43 Å<br>90˚, 90˚, 90˚	47.57, 85.48, 116.79 Å<br>90˚, 90˚, 90˚
**Data collection statistics**		
Resolution limits (outer shell) (Å)	39.6-1.16 (1.19-1.16)	39.2-1.24 (1.27-1.24)
No: of observed reflections (outer shell)	872663 (26507)	1382457 (98249)
No: of unique reflections (outer shell)	80496 (4443)	131248 (9237)
Completeness (outer shell)	96.0 (72.2)	96.9 (93.1)
CC1/2 (outer shell)	99.9 (60.6)	99.7 (68.4)
R_{sym}^{a} (outer shell) (%)	7.1 (168.9)	9.7 (186.2)
Mean I/σ(I) (outer shell)	16.4 (1.4)	11.6 (1.5)
**Refinement statistics**		
Resolution limits (Å)	39.6-1.16	35.4-1.24
Number of reflections (%)	80457 (96.0)	131235 (96.9)
Reflections used for R_{free}	4023	6563
R_{factor}^{b} (%)	14.9	17.7
R_{free} (%)	16.6	21.5
Model contents (average B($Å^2$))		
Protein atoms	2041 (17.3)	4032 (27.2)
Ligand	0	0
Ion/buffer	17 (25.8)	14 (29.8)
Water molecules	302 (34.1)	467 (38.4)
RMS deviations	0.004	0.005
Bond length (Å)	0.82	0.78
Bond angle (°)		
Ramachandran (favored (%)/outliers)	98/0	98/0

[a] $R_{sym} = \Sigma|I_{avg}-I_i|/\Sigma I_i$

[b] R factor = $\Sigma|F_p - F_{pcalc}|/\Sigma F_p$, where F_p and F_{pcalc} are the observed and calculated structure factors; R_{free} is calculated with 5% of the data.

	MHT077^{E47A} (PDB: 9PIR)	EV-MHT043-5 (PDB: 9PJB)
**Crystallographic parameters**		
Space group	$P2_1$	$P2_1$
Unit-cell dimensions	62.21, 63.78, 74.44 Å<br>90˚, 113.6˚, 90˚	47.38, 81.18, 79.92 Å<br>90˚, 106.1˚, 90˚
**Data collection statistics**		
Resolution limits (outer shell) (Å)	37.07-1.46 (1.50-1.46)	39.4-1.77 (1.82-1.77)
No: of observed reflections (outer shell)	493309 (36079)	344142 (23491)
No: of unique reflections (outer shell)	89753 (6390)	56203 (4091)
Completeness (outer shell)	96.7 (94.2)	99.0 (97.2)
CC1/2 (outer shell)	99.9 (59.6)	99.8 (54.3)
R_{sym}^{a} (outer shell) (%)	7.5 (148.3)	8.6 (188.0)
Mean I/σ(I) (outer shell)	17.02 (1.6)	13.3 (1.6)
**Refinement statistics**		
Resolution limits (Å)	37.1-1.46	39.4-1.77
Number of reflections (%)	89733 (96.9)	56156 (99.0)
Reflections used for R_{free}	4487	2806
R_{factor}^{b} (%)	16.7	16.2
R_{free} (%)	20.3	21.9
Model contents (average B(Å^2))		
Protein atoms	3971 (23.8)	4288 (35.7)
Ligand	0	0
Ion/bugger	0	14 (53.7)
Water molecules	423 (35.6)	349 (43.8)
RMS deviations	0.005	0.003
Bond length (Å)	0.78	0.57
Bond angle (°)		
Ramachandran (favored (%)/outliers)	98/0	98/0

[a] $R_{sym} = \Sigma|I_{avg}-I_i|/\Sigma I_i$

[b] R factor = $\Sigma|F_p - F_{pcalc}|/\Sigma F_p$, where F_p and F_{pcalc} are the observed and calculated structure factors; R_{free} is calculated with 5% of the data.

Table S2. MSR-DSC analyses of MHT077.

Scan rate (°C/min)	Total Area (kJ/mol)[a]	$T_{m,app}$ (°C) [b]
0.2	521	85.7
0.4	604	87.0
0.8	639	88.4
1.6	726	90.0
3.2	723	91.7

[a] Area under DSC thermogram and [b] Apparent T_m of enzyme at each scan rate.

Table S3. MSR-DSC analyses of PET46.

Scan rate (°C/min)	Total Area (kJ/mol)	$T_{m,app}$ (°C)
0.2	763	78.8
0.4	842	80.5
0.8	823	82.0
1.6	821	83.7
3.2	881	85.4

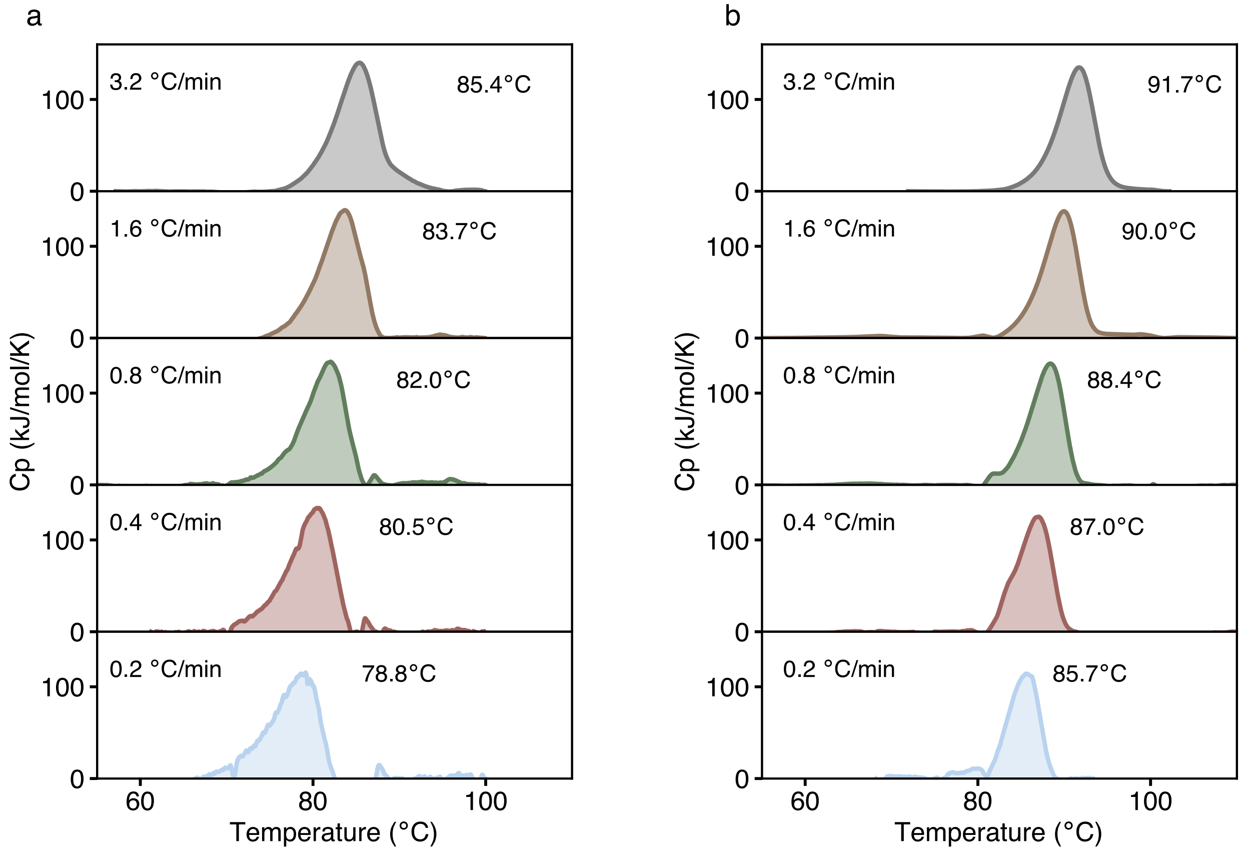

Figure S7: Enzyme $T_{m.app}$ values from MSR-DSC thermograms are dependent on temperature scan rate. DSC thermograms for (a) PET46, and (b) MHT077 recorded with the indicated temperature scan rates. The profiles are consistent with a native-to-denatured model and an irreversible unfolding mechanism. Total area shaded under curves are shown in Tables S2 and 3. Each $T_{m,app}$ is shown inset.

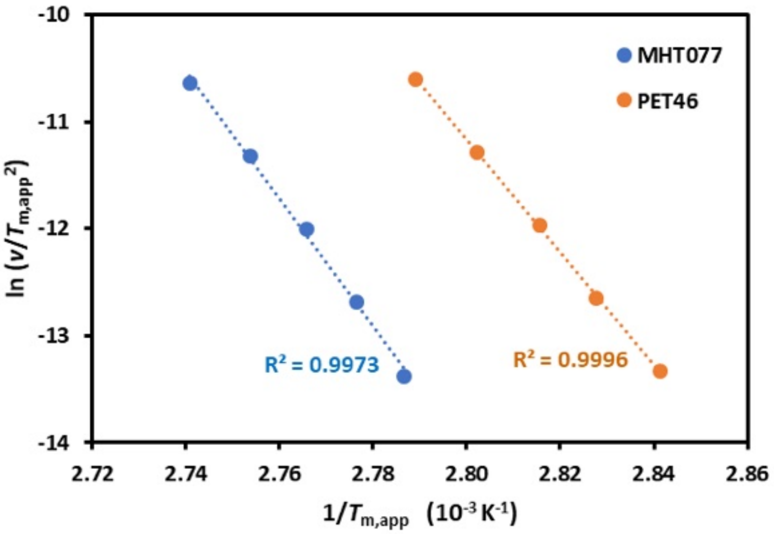

Figure S8: Linear Kissinger plots of VSR-DSC fits for MHT077 and PET46, consistent with an irreversible, native-to-denatured kinetic model.

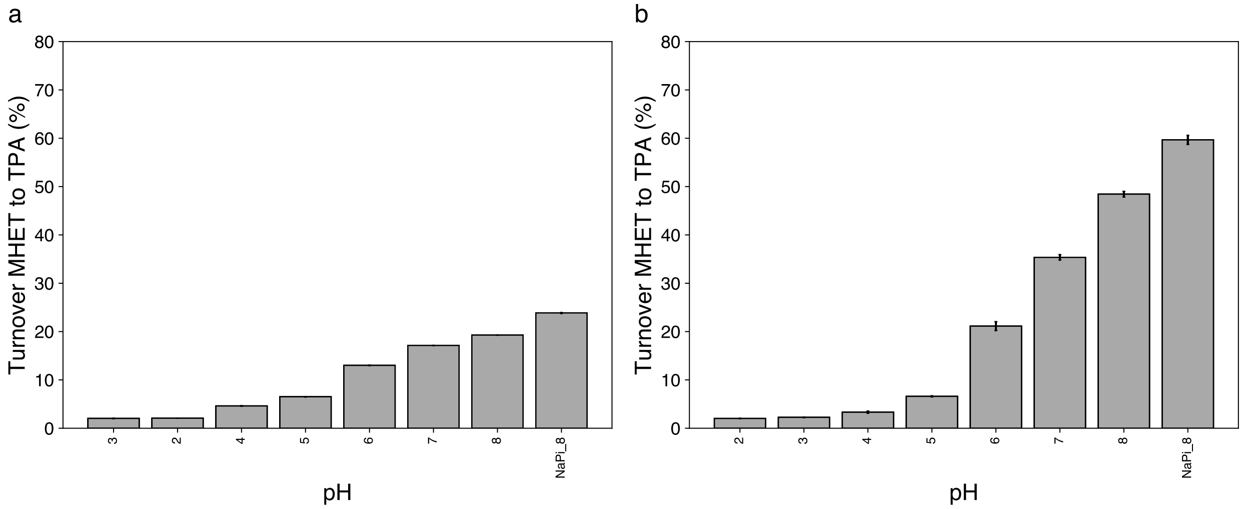

Figure S9: Activity of PET46 (a) and MHT077 (b) at single unit pH intervals across the range of pH 2-8 (1 hour reaction, 65 °C). The Britton-Robinson buffer was used to prepare each reaction condition. For comparison purposes a standard 100 mM sodium phosphate pH 8 condition was included.

Structure-based site-saturation engineering of MHT077

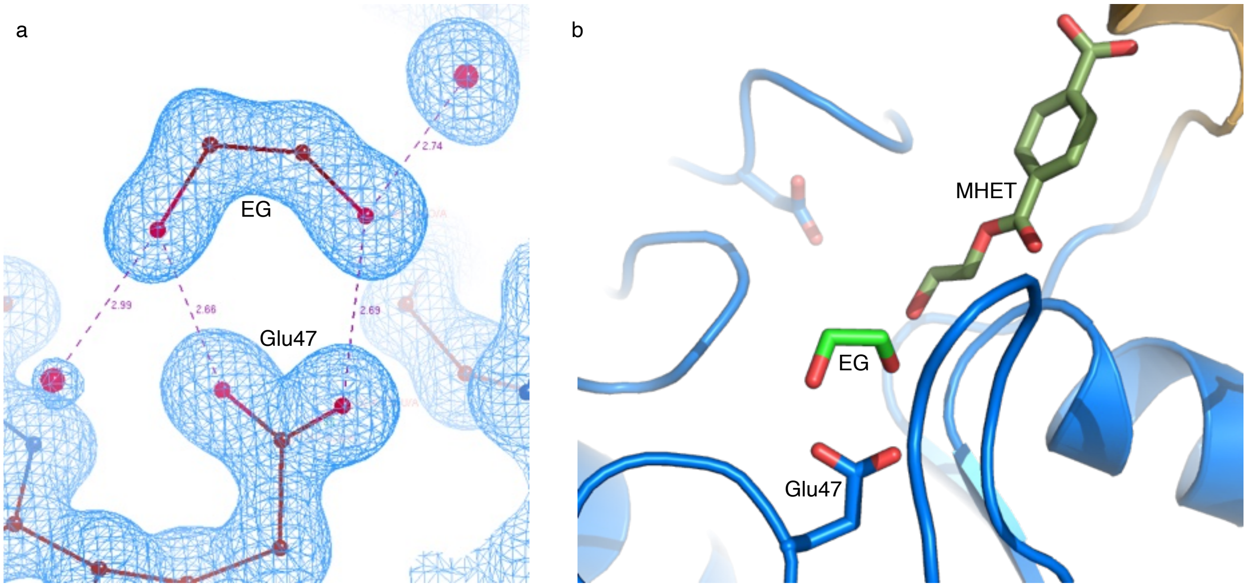

Figure S10. Occupied active site of MHT077. (a) The figure shows a $2F_o$-F_c electron density map contoured at 0.5 e^-/A^3 revealing a well-defined ethylene glycol (EG) molecule sitting within the active site pocket, and in close proximity to residue Glu47. (b) The position of the bound ethylene glycol molecule is shown relative to Glu47 and the approximate position of a manually docked MHET substrate.

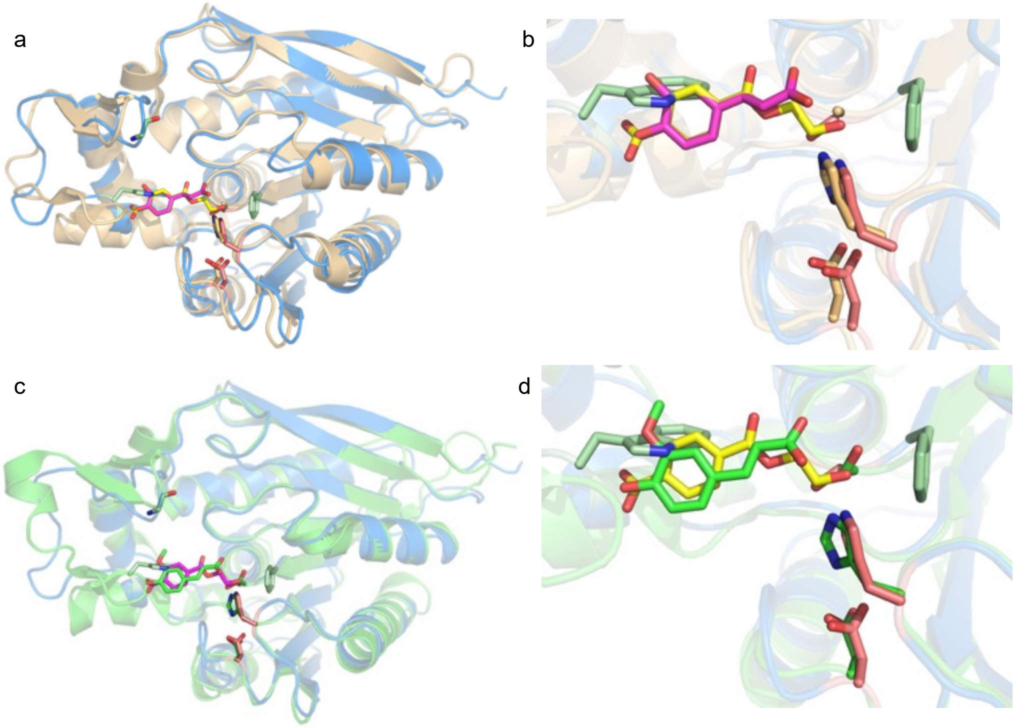

Figure S11. Comparison with homologous crystal structures. (a) Superposition of MHT077 in blue with docked MHET in yellow, and cinnamoyl esterase (3S2Z)[9] in light orange with bound caffeic acid in magenta. Equivalent active site residues in each structure are shown as sticks. (b) Close up comparison of the active site regions demonstrates that docking MHET onto the aromatic portion of caffeic acid results in a steric clash with MHT077 residue Trp148. (c) Superposition of MHT077 (blue) to ferulic acid esterase (7Z2U)[10] in green, with bound ferulate also in green. (d) The bound ferulate ligand of ferulic acid esterase adopts a similar position to caffeic acid in cinnamoyl esterase, also sterically incompatible with docking MHET in proximity to Trp148.

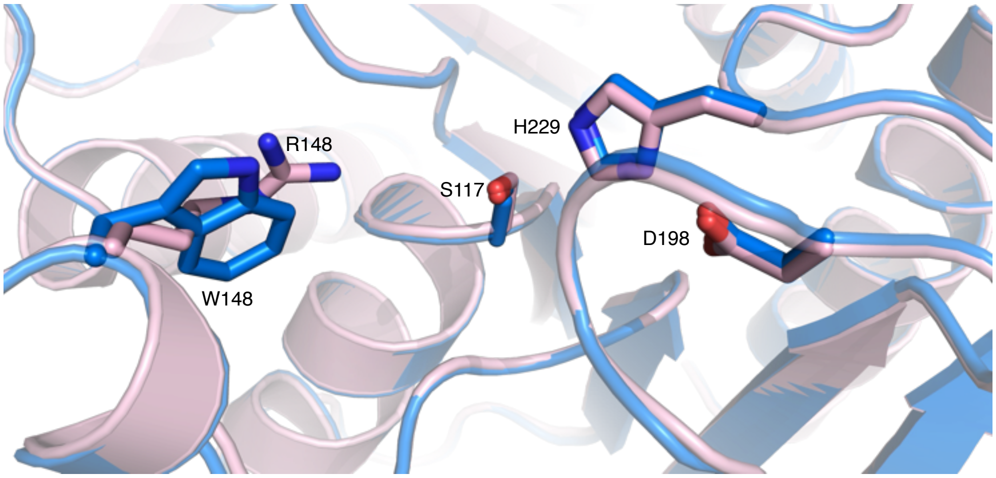

Figure S12. Comparison of parent MHT077 and MHT077^{W148R}. A superposition of coordinates from MHT077 (9PIC, blue) and MHT077^{W148R} (9PID, pink) show no significant change in the overall structure beyond the W148R substitution. The active site triad residues in each structure are also rendered as sticks for reference.

Evolution-informed design of MHETase candidates

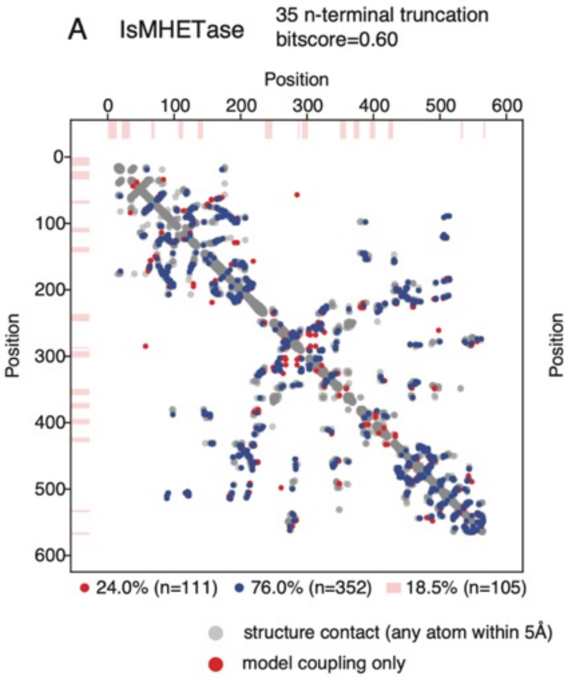

Figure S13. Model quality of *Is*MHETase determined by comparison of the overlap of predicted residue-residue interactions ("couplings") and structural contacts available in PDB structures. (A) *Is*MHETase EVcouplings model derived from a functional n-terminal truncation variant lacking the first 35 amino acids (bitscore parameter, which determines the number of homologs used for modeling was set to 0.6). The top L (length of sequence) predicted residue-residue interactions for each that are at least 5 positions apart in primary sequence are displayed (blue and red dots) and used for percentage calculations below each plot. Structural contacts were determined by the EVcouplings compare tool.[11] Dark blue dot: model-predicted interaction that is also a PDB structural contact. Red dot: model-predicted interaction that is not a PDB structural contact. Gray dots: PDB structural contact defined as residues within 5 angstroms of one another (any atom). Red bar: Positions that are not considered in the model, which indicate many gaps in the multiple sequence alignment used for model inference.

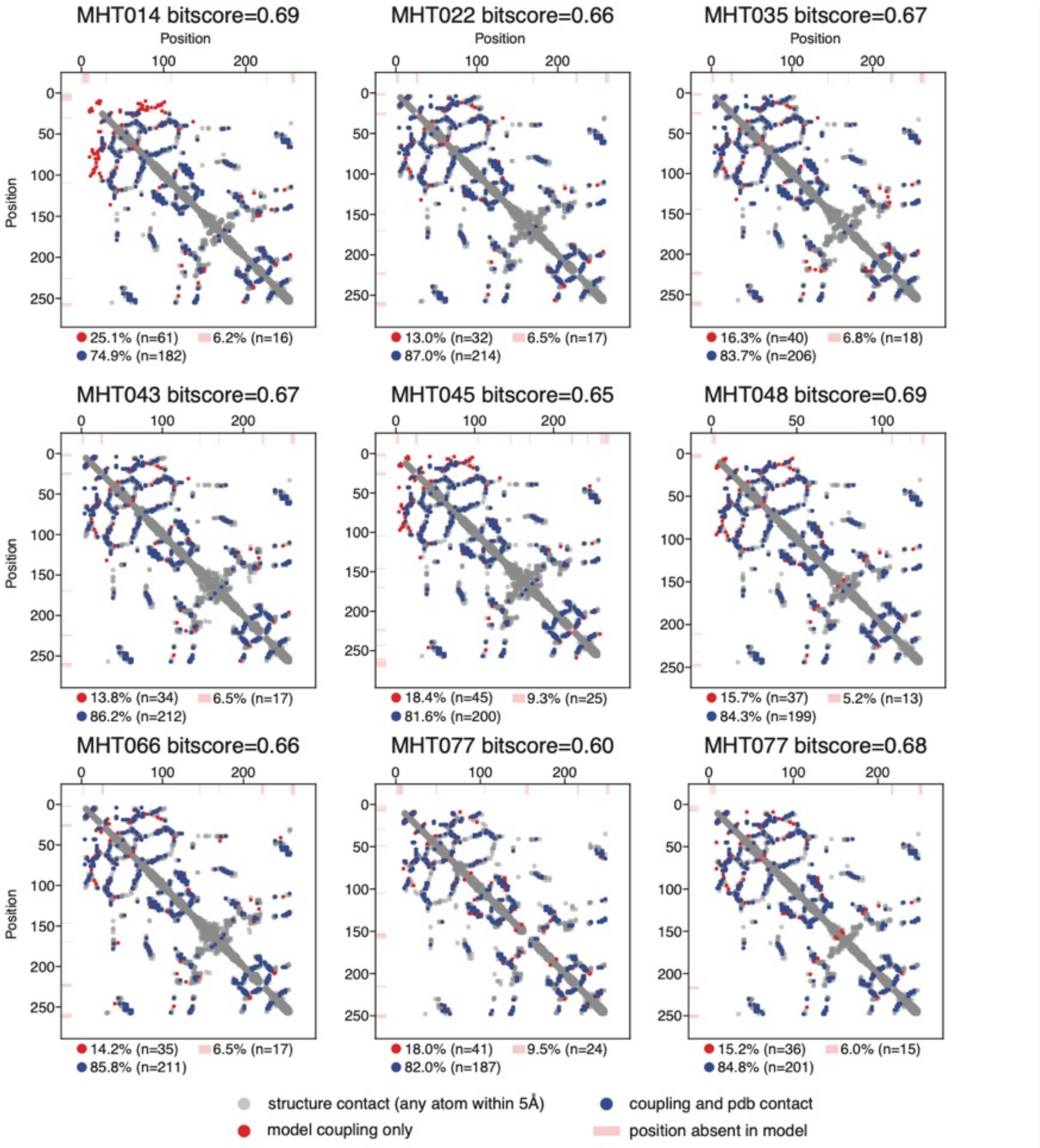

Figure S14. Model quality of natural MHETase scaffolds determined by comparison of the overlap of predicted residue-residue interactions ("couplings") and structural contacts available in PDB structures. The identity of each natural MHETase is indicated above the plot as well as the bitscore (an indicator of how many homologs are used for model inference). The top L (length of sequence) predicted residue-residue interactions that are at least 5 positions apart in primary sequence are displayed (blue and red dots) and used for percentage calculations below each plot. Structural contacts were determined by the EVcouplings compare tool.[11] Dark blue dot: model-predicted interaction that is also a PDB, structural contact. Red dot: model-predicted interaction that is not a PDB structural contact. Gray dots: PDB structural contact defined as residues within 5 angstroms of one another (any atom). Red bar: Positions that are not considered in the model, which indicate many gaps in the multiple sequence alignment used for model inference.

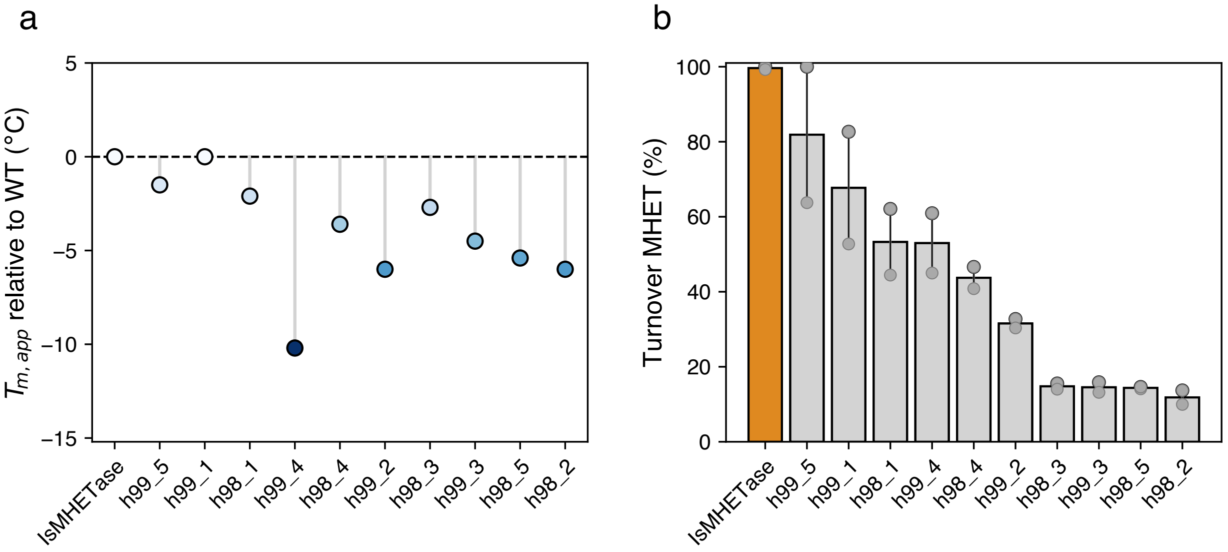

Figure S15: (a) Apparent protein melting temperatures determined by the Sypro-orange dye-binding assay at a temperature ramp rate of 0.3 °C/s (18 °C/min) and (b) MHET hydrolase activity at 50 ºC of 10 EV designs based on *Is*MHETase (WT control shown in orange). Error bars represent the range between biological duplicates. The two sets of EV couplings parameters that were applied to generate the designs (denoted by h98 and h99 respectively) are described in the Methods.

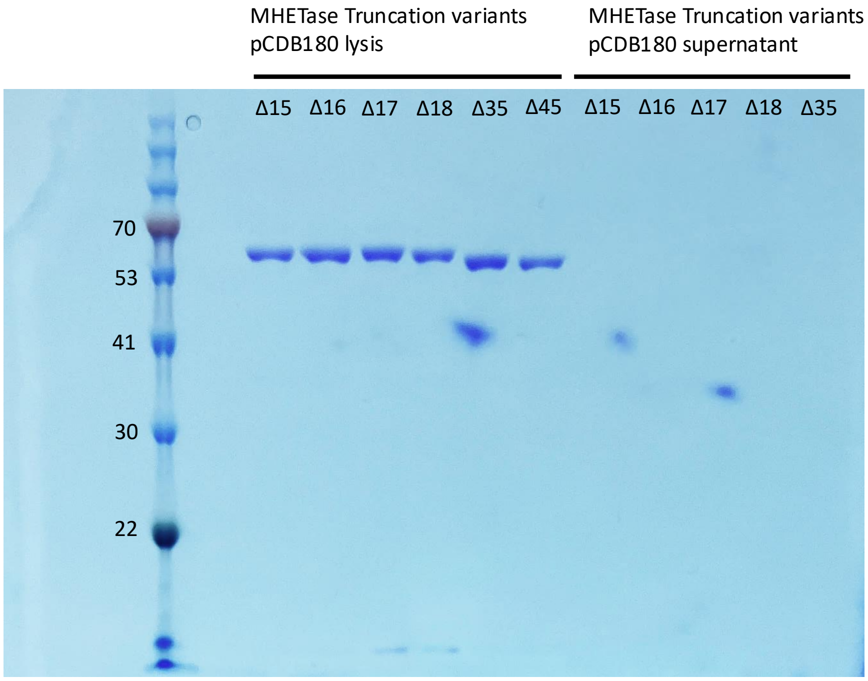

Figure S16. SDS PAGE gel of purified *Is*MHETase proteins with N-terminal truncations. The *Is*MHETase truncations were expressed in pCDB180. Samples of purified *Is*MHETase N-terminal deletion proteins, Δ15, Δ16, Δ17, Δ35, and Δ45, with the supernatants from expression of Δ16-45 shown tor the right alongside marker (lane 1 – molecular weights denoted). Samples were boiled in Laemmli buffer at 95 °C prior to loading on the gel. The proteins were only observed in the lysates samples.

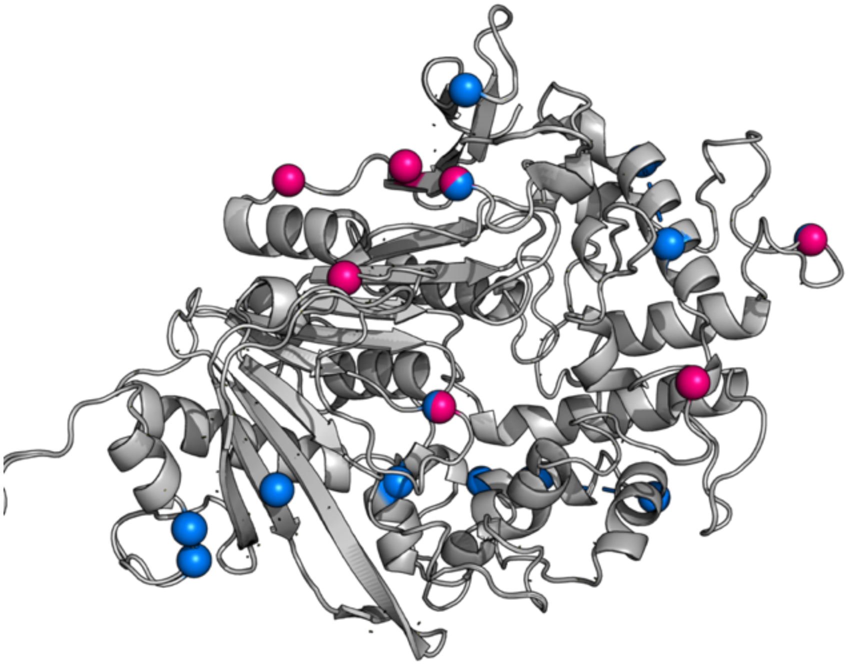

Figure S17: Overlayed AlphaFold3 predicted structures of EV-design-h99-1 and EV-design-h99-5 based on *Is*MHETase. The 9 mutations introduced by the EVcouplings method into design 1 are shown as pink spheres, and the 12 mutations of design 5 shown as blue spheres. Overlapping mutation sites are shown as split-colored blue and pink spheres. The SI Dataset contains the individual protein sequences.

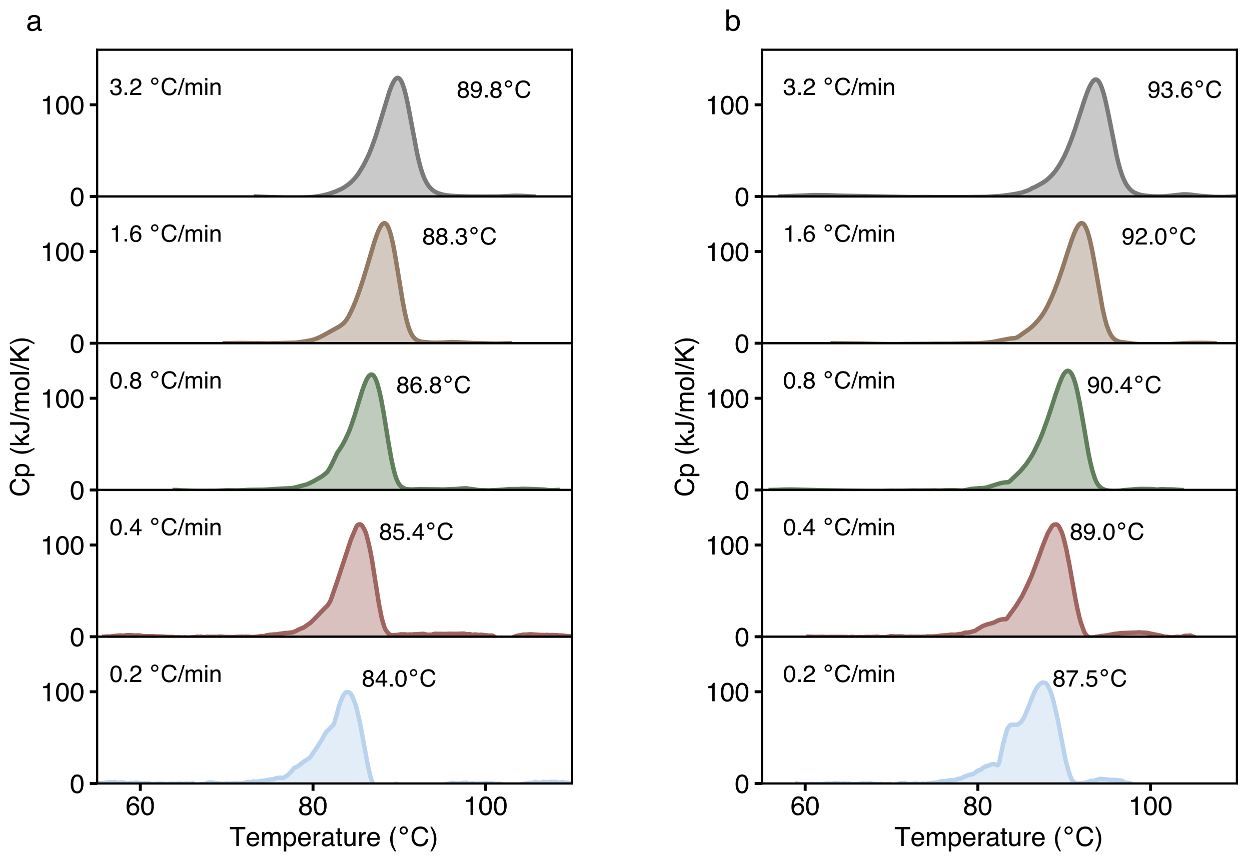

Figure S18: Enzyme kinetic stability analysis from MSR-DSC. DSC thermograms for (a) $MHT077^{LFK}$ and (b) $MHT077^{FFN}$, recorded with the indicated temperature scan rates. The profiles are consistent with a native to denatured model and an irreversible unfolding mechanism. Total area shaded under curves are shown in Tables S4-5. Each $T_{m,app}$ value is shown inset.

Table S4. Kinetic stability analyses from MSR-DSC thermograms of MHT077^{LFK}.

Scan rate (°C/min)	Total Area (kJ/mol)	$T_{m,app}$ (°C)
0.2	475	84.0
0.4	632	85.4
0.8	636	86.8
1.6	646	88.3
3.2	651	89.8

Table S5. Kinetic stability analyses from MSR-DSC thermograms of MHT077^{FFN}.

Scan rate (°C/min)	Total Area (kJ/mol)	$T_{m,app}$ (°C)
0.2	669	87.5
0.4	694	89.0
0.8	686	90.4
1.6	684	92.0
3.2	691	93.6

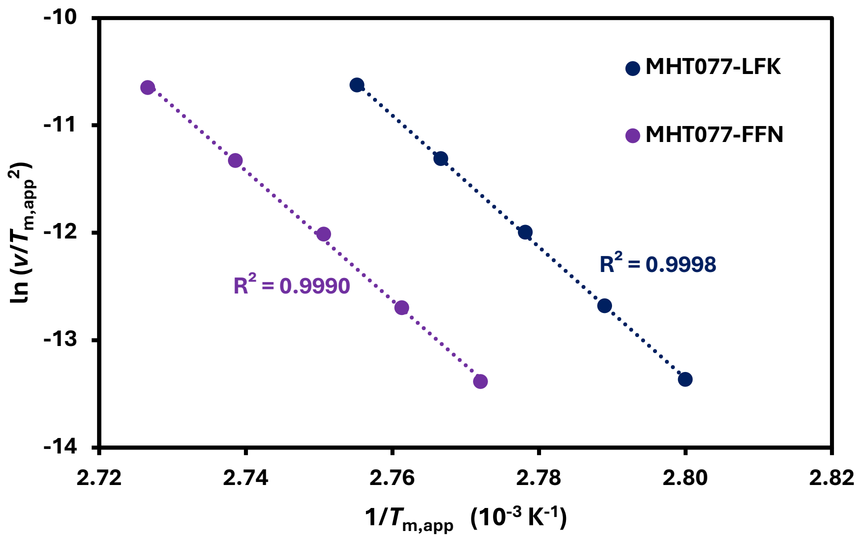

Figure S19. Linear Kissinger plots of MSR-DSC fits for MHT077 variants, MHT077^{LFK} and MHT077^{FFN}, consistent with an irreversible, native-to-denatured kinetic model.

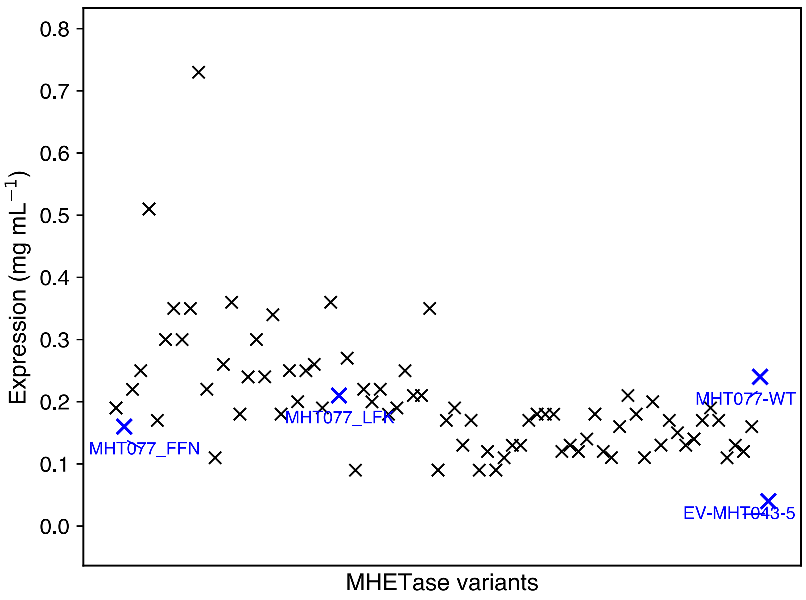

Figure S20. Scatter plot showing expression titers of MHETase variants with the top-performing MHETase variants, MHT077-WT, MHT077-LFK, MHT077-FFN and EV-MHT043-5 marked as blue crosses. Expression values are plotted in mg/mL, where all variants were expressed as described in the Enzyme production and purification section of the Experimental Methods.

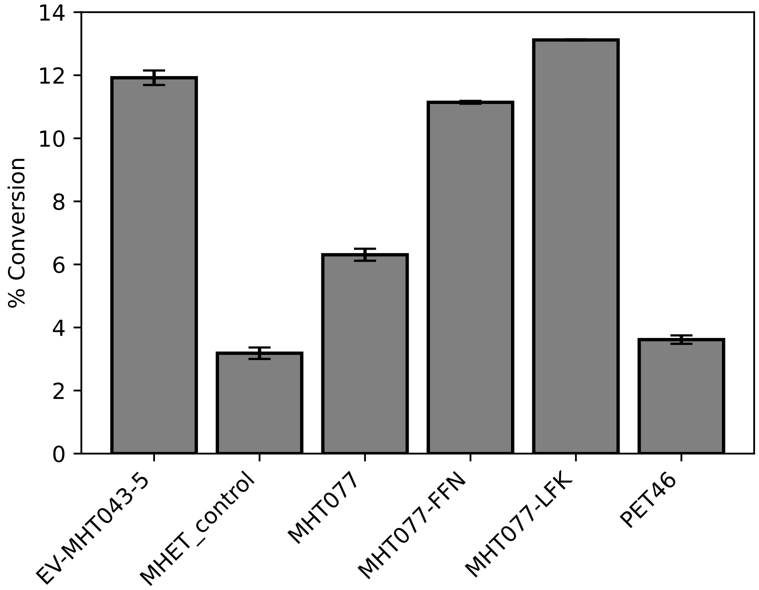

Figure S21. MHETase activity of PET46, MHT077-WT, MHT077 variants, and EV-MHT043 at 65 ºC in small-scale reactions (20 mM MHET in 0.1 M NaPi pH 8, 65 ºC), expressed as percent MHET conversion. Bars represent mean values with error bars centered on the range of two biological replicates. An autohydrolysis substrate control of 20 mM MHET in 100 mM sodium phosphate pH 8 is shown labelled as 'MHET_control'.

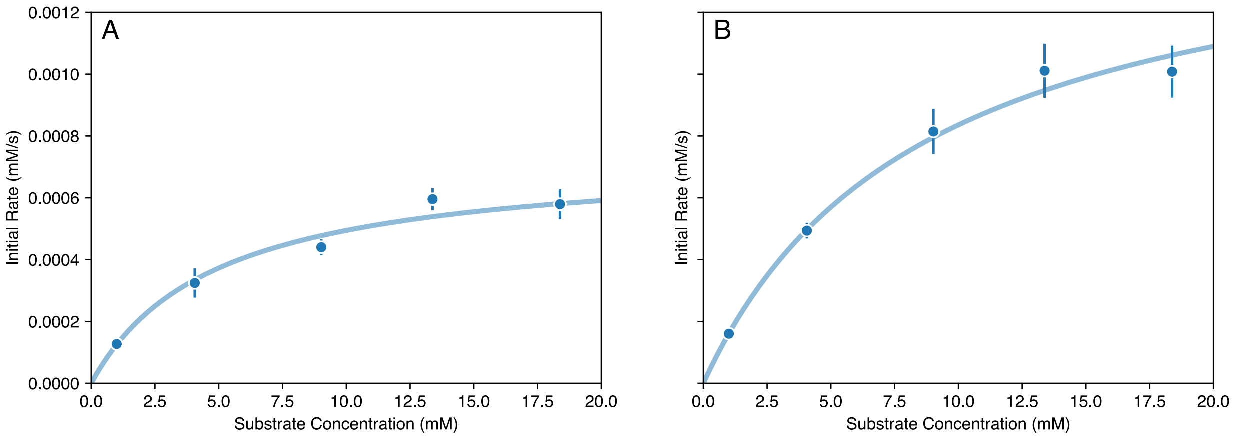

Figure S22. Kinetics of MHT077 WT and MHT077^{LFK} measured at 65 ºC in 0.1 M sodium phosphate pH 8. Initial rates of TPA production, calculated using linear regression, are shown plotted against MHET concentration (0-20 mM). A Michaelis-Menten kinetics model was applied to fit each curve and extract the k_{cat} and K_m values shown in Table S6. Error bars represent mean values with error bars centered on the range of two replicates.

Table S6. Kinetic parameters of WT-MHT077 and the MHT077^{LFK} variant at 65 ºC.

Enzyme	Mutations relative to WT	k_{cat} (s^{-1})	K_m (mM)
MHT077	-	2.45 ± 0.20	4.85 ± 0.70
MHT077^{LFK}	F116L, W148F, G162K	5.22 ± 0.34	8.73 ± 0.94

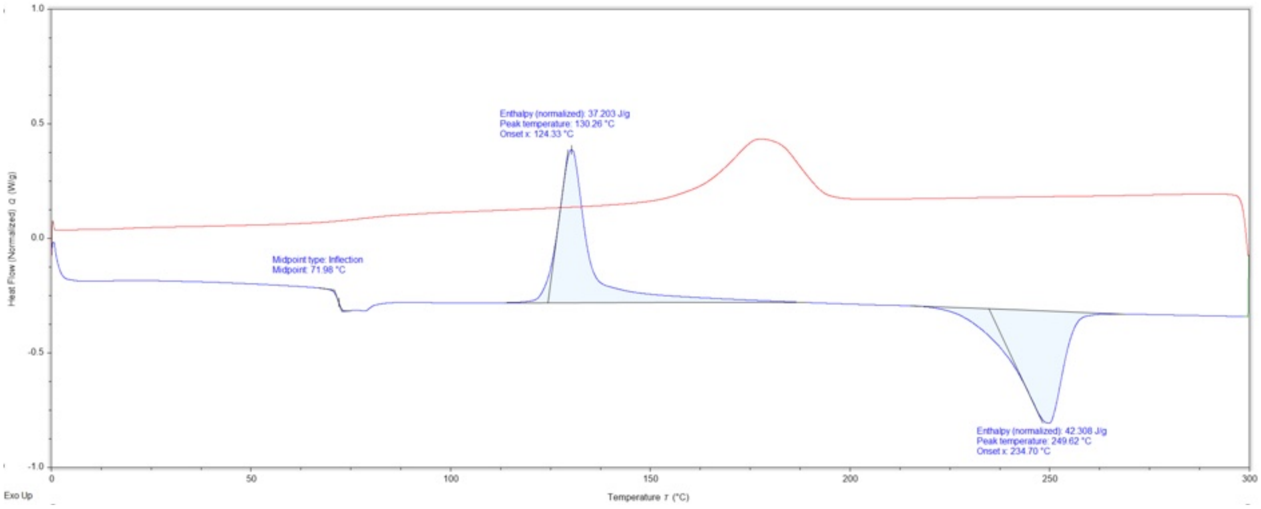

Figure S23. Differential scanning calorimetry (DSC) thermograms of ground thermoform PET substrate. Exothermic is up, endothermic is down. Percent crystallinity was calculated via Equation S1 (see Methods) and is presented in **Table S7**.

Table S7: Thermal properties and crystallinity of the three replicates of amorphous ground thermoform PET shown in **Figure S23**.

Sample	T_g (°C)	Heat of Melting (J/g)	Heat of Cold Crystallization (J/g)	Percent Crystallinity

**1**	72.5	42.3	37.2	3.64
**2**	72.5	40.1	31.8	5.94
**3**	72.3	42.2	38.0	3.03
**Average**	72.4	41.6 ± 1.22	35.7 ± 3.35	4.21 ± 1.53

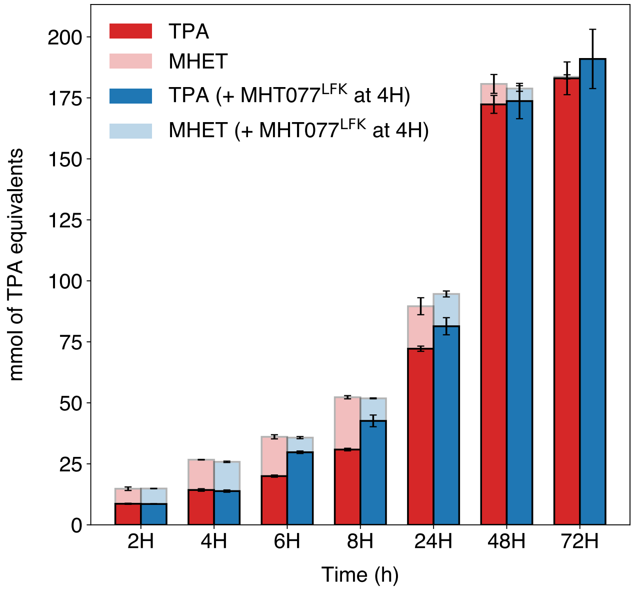

Figure S24: Yields of pH-controlled deconstructions of PET (37.5 g in 0.25 L) with LCC^{ICCG} (red) and a two-enzyme cascade of LCC^{ICCG} and MHETase variant, MHT77^{LFK} (blue). TPA and MHET analytes were determined by UHPLC. Error bars are centered on the average of two duplicate reactors.

References

(1) Gilchrist, C. L. M.; Mirdita, M.; Steinegger, M. Multiple Protein Structure Alignment at Scale with FoldMason. bioRxiv August 1, 2024, p 2024.08.01.606130. https://doi.org/10.1101/2024.08.01.606130.

(2) Abramson, J.; Adler, J.; Dunger, J.; Evans, R.; Green, T.; Pritzel, A.; Ronneberger, O.; Willmore, L.; Ballard, A. J.; Bambrick, J.; Bodenstein, S. W.; Evans, D. A.; Hung, C.-C.; O'Neill, M.; Reiman, D.; Tunyasuvunakool, K.; Wu, Z.; Žemgulytė, A.; Arvaniti, E.; Beattie, C.; Bertolli, O.; Bridgland, A.; Cherepanov, A.; Congreve, M.; Cowen-Rivers, A. I.; Cowie, A.; Figurnov, M.; Fuchs, F. B.; Gladman, H.; Jain, R.; Khan, Y. A.; Low, C. M. R.; Perlin, K.; Potapenko, A.; Savy, P.; Singh, S.; Stecula, A.; Thillaisundaram, A.; Tong, C.; Yakneen, S.; Zhong, E. D.; Zielinski, M.; Žídek, A.; Bapst, V.; Kohli, P.; Jaderberg, M.; Hassabis, D.; Jumper, J. M. Accurate Structure Prediction of Biomolecular Interactions with AlphaFold 3. *Nature* **2024**, *630* (8016), 493–500. https://doi.org/10.1038/s41586-024-07487-w.

(3) Sievers, F.; Wilm, A.; Dineen, D.; Gibson, T. J.; Karplus, K.; Li, W.; Lopez, R.; McWilliam, H.; Remmert, M.; Söding, J.; Thompson, J. D.; Higgins, D. G. Fast, Scalable Generation of High-quality Protein Multiple Sequence Alignments Using Clustal Omega. *Molecular Systems Biology* **2011**, *7* (1), 539. https://doi.org/10.1038/msb.2011.75.

(4) Kozlov, A. M.; Darriba, D.; Flouri, T.; Morel, B.; Stamatakis, A. RAxML-NG: A Fast, Scalable and User-Friendly Tool for Maximum Likelihood Phylogenetic Inference. *Bioinformatics* **2019**, *35* (21), 4453–4455. https://doi.org/10.1093/bioinformatics/btz305.

(5) Buchfink, B.; Xie, C.; Huson, D. H. Fast and Sensitive Protein Alignment Using DIAMOND. *Nat Methods* **2015**, *12* (1), 59–60. https://doi.org/10.1038/nmeth.3176.

(6) Seemann, T. Prokka: Rapid Prokaryotic Genome Annotation. *Bioinformatics* **2014**, *30* (14), 2068–2069. https://doi.org/10.1093/bioinformatics/btu153.

(7) Gilchrist, C. L. M.; Chooi, Y.-H. Clinker & Clustermap.Js: Automatic Generation of Gene Cluster Comparison Figures. *Bioinformatics* **2021**, *37* (16), 2473–2475. https://doi.org/10.1093/bioinformatics/btab007.

(8) Marchler-Bauer, A.; Bo, Y.; Han, L.; He, J.; Lanczycki, C. J.; Lu, S.; Chitsaz, F.; Derbyshire, M. K.; Geer, R. C.; Gonzales, N. R.; Gwadz, M.; Hurwitz, D. I.; Lu, F.; Marchler, G. H.; Song, J. S.; Thanki, N.; Wang, Z.; Yamashita, R. A.; Zhang, D.; Zheng, C.; Geer, L. Y.; Bryant, S. H. CDD/SPARCLE: Functional Classification of Proteins via Subfamily Domain Architectures. *Nucleic Acids Res* **2017**, *45* (D1), D200–D203. https://doi.org/10.1093/nar/gkw1129.

(9) Lai, K.-K.; Stogios, P. J.; Vu, C.; Xu, X.; Cui, H.; Molloy, S.; Savchenko, A.; Yakunin, A.; Gonzalez, C. F. An Inserted α/β Subdomain Shapes the Catalytic Pocket of Lactobacillus Johnsonii Cinnamoyl Esterase. *PLOS ONE* **2011**, *6* (8), e23269. https://doi.org/10.1371/journal.pone.0023269.

(10) Kasmaei, K. M.; Kalyani, D. C.; Reichenbach, T.; Jiménez-Quero, A.; Vilaplana, F.; Divne, C. Crystal Structure of the Feruloyl Esterase from Lentilactobacillus Buchneri Reveals a Novel Homodimeric State. *Front. Microbiol.* **2022**, *13*. https://doi.org/10.3389/fmicb.2022.1050160.

(11) Hopf, T. A.; Green, A. G.; Schubert, B.; Mersmann, S.; Schärfe, C. P. I.; Ingraham, J. B.; Toth-Petroczy, A.; Brock, K.; Riesselman, A. J.; Palmedo, P.; Kang, C.; Sheridan, R.; Draizen, E. J.; Dallago, C.; Sander, C.; Marks, D. S. The EVcouplings Python Framework for Coevolutionary Sequence Analysis. *Bioinformatics* **2019**, *35* (9), 1582–1584. https://doi.org/10.1093/bioinformatics/bty862.
